# The differences in essential facial areas for impressions between humans and deep learning models: An eye‐tracking and explainable AI approach

**DOI:** 10.1111/bjop.12744

**Published:** 2024-10-25

**Authors:** Takanori Sano, Jun Shi, Hideaki Kawabata

**Affiliations:** ^1^ Graduate School of Human Relations Keio University Minato‐ku Tokyo Japan; ^2^ Faculty of Letters Keio University Minato‐ku Tokyo Japan

**Keywords:** deep learning, explainable AI, eye‐tracking, facial impressions, geometric morphometrics

## Abstract

This study explored the facial impressions of attractiveness, dominance and sexual dimorphism using experimental and computational methods. In Study 1, we generated face images with manipulated morphological features using geometric morphometrics. In Study 2, we conducted eye tracking and impression evaluation experiments using these images to examine how facial features influence impression evaluations and explored differences based on the sex of the face images and participants. In Study 3, we employed deep learning methods, specifically using gradient‐weighted class activation mapping (Grad‐CAM), an explainable artificial intelligence (AI) technique, to extract important features for each impression using the face images and impression evaluation results from Studies 1 and 2. The findings revealed that eye‐tracking and deep learning use different features as cues. In the eye‐tracking experiments, attention was focused on features such as the eyes, nose and mouth, whereas the deep learning analysis highlighted broader features, including eyebrows and superciliary arches. The computational approach using explainable AI suggests that the determinants of facial impressions can be extracted independently of visual attention.

## BACKGROUND

During social interactions, people can read various social information from others' faces. The face is a crucial element in impression formation, and numerous studies have been conducted on the components of facial impressions, such as attractiveness. For example, facial averageness and symmetry are factors of facial attractiveness (Grammer & Thornhill, 1994; Langlois & Roggman, 1990). Facial attractiveness is influenced by components of the face such as the eyes, mouth, and nose (Terry & Davis, 1976), as well as skin luminance (Russell, 2003) and contrast (Russell, 2009). Another factor related to facial attractiveness is sexual dimorphism, or masculinity for male faces and femininity for female faces. Faces that emphasize sexual dimorphism are more attractive (Perrett et al., 1998), and sexual dimorphism is associated with morphological features determined by sex hormones (Little et al., 2011; Rhodes, 2006; Thornhill & Gangestad, 1999). In the female face, oestrogen suppresses bone growth, resulting in rounded cheeks and lips. In the male face, testosterone develops cheekbones and jawlines, elevates brow muscles, projects the centre of the face forward and increases the length of the face from cheek to jaw. Studies have reported differences in same‐ and opposite‐sex ratings (Little et al., 2001, 2002).

Dominance is one of the impressions associated with attractiveness and sexual dimorphism (Perrett et al., 1998). It is related to facial features that indicate physical strength or weakness and is an important dimension in social judgements, such as of threat (Oosterhof & Todorov, 2008). Faces that emphasize impressions of dominance have prominent morphological features, such as lifted eyes and eyebrows, that resemble the facial structure during the expression of anger (Montepare & Dobish, 2003). Factors that determine these facial impressions have been studied mainly with experimental and computational methods (Adolphs et al., 2016; Said & Todorov, 2011).

In experimental research, only certain facial features are manipulated based on the hypothesis, with other features being controlled to investigate the relation between specific facial features and facial impressions. Although this method limits the investigation to the hypothesized facial features, it is excellent for examining hypothesized factors under strict control. Experimental studies using eye‐tracking have investigated the features perceivers pay attention to when evaluating facial impressions. For example, when presented with facial stimuli, people tend to make eye movements towards the eyes, mouth and nose (Barton et al., 2006; Walker‐Smith et al., 1977). These areas are also fixated upon when evaluating the age and attractiveness of a face (Kwart et al., 2012). The nose is fixated on longer when evaluating the attractiveness of facial images (Zhang et al., 2017). The mouth has a higher fixation density in happiness judgements than in trustworthiness judgements of happy faces (Calvo et al., 2019); however, dominance and trustworthiness rating tasks show no differences in fixation (Hermens et al., 2018). Thus, the influence of the evaluation task is also known. Additionally, fixation tendencies are influenced by sex and individual differences (Dindaroğlu et al., 2017; Garza & Byrd‐Craven, 2023; Leder et al., 2010). For example, attractive faces are observed for longer, especially female perceivers viewing female faces (Leder et al., 2010), and women with higher marital orientation pay increased visual attention to masculine faces (Garza & Byrd‐Craven, 2023).

Computational approaches (Sutherland et al., 2017) have been proposed to minimize prior knowledge bias and consider the influence of all analysed facial features. Example approaches include geometric morphometrics (Mitteroecker et al., 2015; Nakamura et al., 2020; Valenzano et al., 2006; Windhager et al., 2011, 2018), which can generate various faces by manipulating facial morphological features, and deep learning methods, which can analyse the relation between facial impressions and image features using facial image data (Sano, 2022, 2023; Sano & Kawabata, 2023).

Using geometric morphometrics, Nakamura et al. (2020) showed that facial features, such as raised corners of the mouth and eyebrows, corresponded to valence. Windhager et al. (2011) demonstrated that very attractive, tall men had longer and narrower chins, wider and fuller lips, rounder faces and thicker eyebrows. They also showed that dominance and masculinity are associated with a round face, thick eyebrows and a prominent chin. In a later study, they reported an asymmetric inverse U‐shaped relation between body fat percentage and attractiveness (Windhager et al., 2018). Masculine facial shapes are characterized by a wide interorbital distance, a broad nose, thin lips and a large, massive lower face (Mitteroecker et al., 2015). In women, attractive face shapes and sexual dimorphism are similar in the upper part of the face but markedly different in the jaw and chin (Valenzano et al., 2006).

Using deep learning, models that predict facial beauty and attractiveness have emerged (Saeed & Abdulazeez, 2021). With the recent development of explainable AI (a technique producing AI inference outputs in a form interpretable to humans), researchers are starting to investigate facial impression factors. For example, convolutional neural networks (CNNs) and their hidden layer visualization methods have shown that sexual dimorphism features are related to facial attractiveness (Sano, 2022, 2023). A recent study analysed facial attractiveness by considering morphological and other features using geometric morphometrics and deep learning (Sano & Kawabata, 2023).

However, hypothesis‐driven experimental methods cannot readily be used to investigate many facial features other than those hypothesized, and data‐driven computational methods cannot confirm whether the extracted results are consistent with actual impression perception. Therefore, although several studies have involved validation tests using face images generated by geometric morphometrics to evaluate actual impressions (Nakamura et al., 2020; Sano & Kawabata, 2023), no study has fully determined the face areas on which evaluators tend to focus. Additionally, while impression perception is influenced by the sex of the perceiver and the face image (Little et al., 2001, 2002), the details of this difference are also unclear. Furthermore, deep learning requires a large dataset of face images with face impression score labels; because such datasets have few images, deep learning analysis of dominance and sexual dimorphism has not been fully explored. Finally, because there is only one label assigned to a face image in many datasets, the characteristics of the labelled raters have not been sufficiently examined.

Oosterhof and Todorov (2008) reported that valence and trustworthiness are important dimensions of facial impressions, alongside attractiveness, dominance and sexual dimorphism. Other studies have also highlighted that valence and trustworthiness correlate with attractiveness (Li & Liu, 2021; Todorov et al., 2008). Moreover, trustworthiness and dominance are known to be negatively correlated in female faces (Oh et al., 2020; Sutherland et al., 2015). In this study, we aimed to explore the impact of sexual dimorphism on attractiveness ratings, considering inconsistencies in the reported effects of masculinity and femininity of male faces (DeBruine et al., 2006; Little et al., 2001; Perrett et al., 1998), and the role of dominance impressions as a contributing factor (Perrett et al., 1998).

In this study, we explored the facial impression factors of attractiveness, dominance and sexual dimorphism. In Study 1, we generated face images with manipulated morphological features using geometric morphometrics, aiming to set conditions for experiments regarding the facial features involved in facial impressions. In Study 2, these conditions were tested empirically. Using an eye‐tracking and impression evaluation experiment, we tested whether these features were important to perceivers. In addition, we examined the correspondence with the fixation area during impression evaluation, including computationally extracted facial features, and the relationship between the sex of the image and the participant. In Study 3, we analysed important features for facial impressions by sex of image and raters using a deep learning method. These studies aimed to exploratorily elucidate the relation between the computationally extracted features and actual fixation areas of the perceivers, considering the sex of the image and perceiver.

## STUDY 1: ANALYSIS AND DATA GENERATION USING GEOMETRIC MORPHOMETRICS

First, we used geometric morphometrics to generate facial images highlighting facial features potentially involved in impressions and searched for features corresponding to facial impressions.

### Methods

We used the Chicago Face Database (Ma et al., 2015), which contains various facial impression scores labelled by raters of various nationalities. We used 290 male and 307 female images from the Asian, Latino, Black and White races for which all the impression scores for attractiveness, dominance and sexual dimorphism (masculinity and femininity) were available. For sexual dimorphism, we used masculinity and femininity scores for the male and female images respectively. These scores are assigned an average value for each image evaluated by volunteers of various nationalities on a 7‐point scale from 1 to 7. The mean and standard deviation of the impression scores for the dataset were as follows: For male images, the mean attractiveness score was 3.00 (*SD* = 0.63), the mean dominance score was 3.10 (*SD* = 0.69) and the mean sexual dimorphism score was 4.61 (*SD* = 0.58). For female images, the mean attractiveness score was 3.45 (*SD* = 0.82), the mean dominance score was 2.58 (*SD* = 0.55) and the mean sexual dimorphism score was 4.50 (*SD* = 0.72).

First, we assigned facial landmark points to each face image (Singh et al., 2022). For smooth image generation, we used 52 as the main landmark points for analysis and 87 as semi‐landmarks for imaging smoothing. Second, we minimized the distance from the reference via Procrustes analysis (Kendall, 1977, 1984) and matched the centre of gravity as the reference position of the landmark points. Third, we computed warps from the landmark points using thin‐plate splines (Bookstein, 1989, 1996) and concatenated and smoothed the fragments between landmarks. Warps correspond to changes in facial morphology. We conducted a multivariate regression analysis using warps as the dependent variable and facial impression scores as the independent variable, by sex and by impression. In other words, we constructed a regression model showing the variation of shape information linked to impression scores. We used permutation testing (10,000 permutations) to compute the statistical significance (*p*‐values) of the explained variance for each regression model. Finally, we visualized landmark locations and facial images. We used tpsRelw (Version 1.75) for landmark point standardization (Rohlf,  2015), tpsRegr (Version 1.50) for regression analysis and landmark point visualization (Rohlf, 2015) and tpsSuper (Version 2.06) for imaging (Rohlf, 2015).

### Results and discussion

**FIGURE 1:**
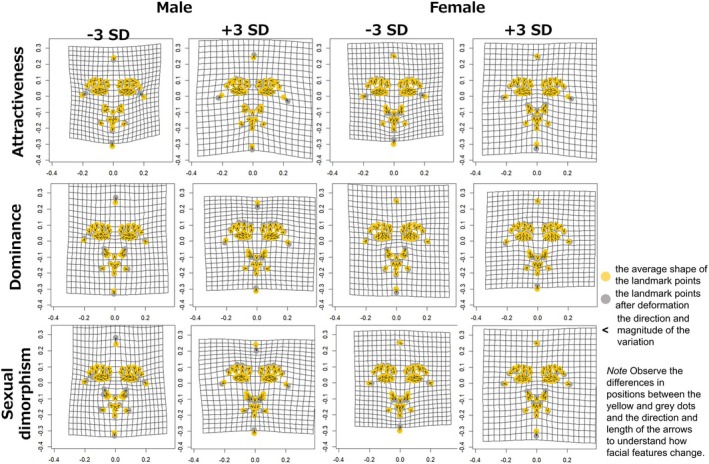
Visualization of landmark points by geometric morphometrics. The upper, middle and lower rows show the change from the average shape of landmark points corresponding to the attractiveness, dominance and sexual dimorphism ratings respectively. Yellow dots indicate landmark points with the average shape, and grey dots indicate landmark points with the shape after deformation. The black arrows indicate the direction of the variation: Low (−3 *SD*) indicates the result when the ratings are manipulated towards −3 *SD*, and High (+3 *SD*) indicates the result when the ratings are manipulated towards +3 *SD*.

**FIGURE 2:**
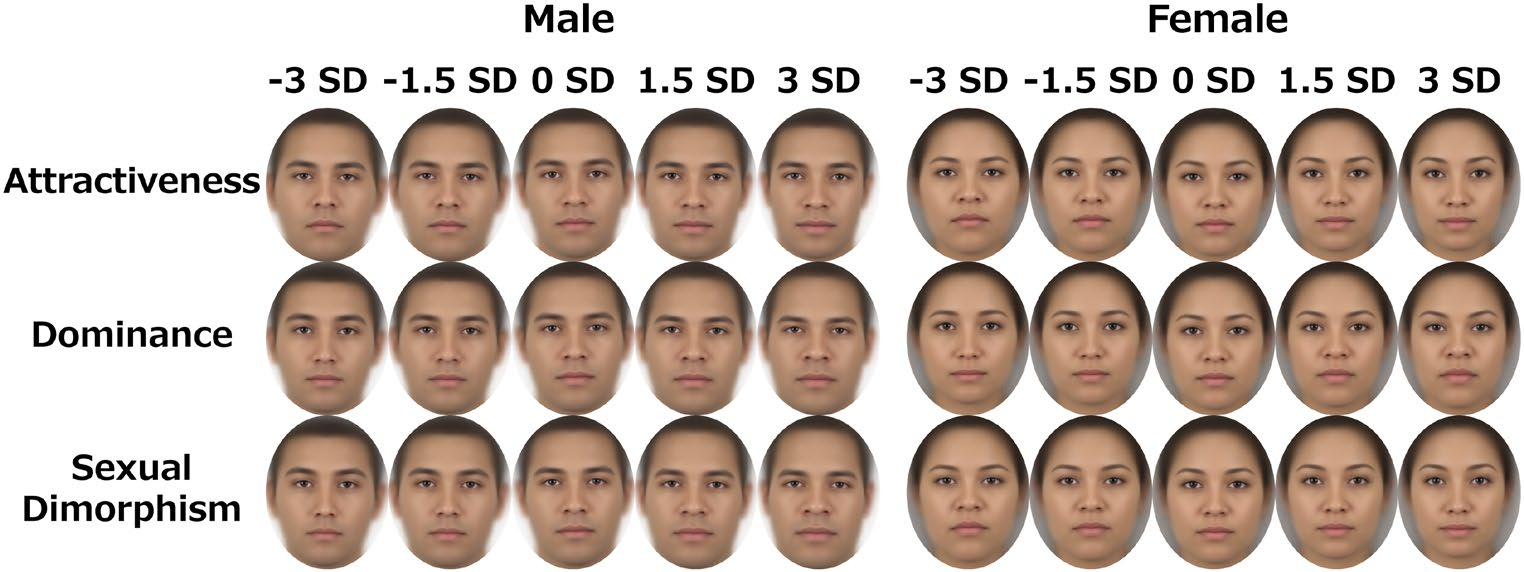
Results of images processed with morphing analysis. The upper, middle and lower rows show the superimposed average face images corresponding to the attractiveness, dominance and sexual dimorphism ratings respectively. Low (−3 *SD*) indicates the result when the ratings are manipulated towards −3 *SD*, and High (+3 *SD*) indicates the result when the ratings are manipulated towards +3 *SD*.

Multivariate regression analysis revealed that the face shape component and facial impression scores were significantly correlated with attractiveness (male and female models: 1.36% and 1.93% of variance explained, respectively, *p* < .001), dominance (male and female models: 2.19% and 1.58% of variance explained, respectively, *p* < .001) and sexual dimorphism (male and female models: 3.07% and 2.10% of variance explained, respectively, *p* < .001). The results of the visualization and imaging (Figures 1 and 2) showed that attractive faces tended to have large eyes, sharply angled contours and elevated eyebrows, regardless of sex. The tendency towards an elevated brow was particularly emphasized for the dominant face. For the sex‐specific faces, the nose and mouth parts were emphasized for the male faces, whereas the facial parts tended to be rounded for the female images. These features were generally consistent with previous data‐driven studies (Nakamura et al., 2020; Nakamura & Watanabe, 2019), suggesting a relation to testosterone and oestrogen, which are involved in masculinity and femininity respectively (Little et al., 2011; Rhodes, 2006; Thornhill & Gangestad, 1999). We set these generated facial features as the experimental hypotheses for the subsequent Study 2.

## STUDY 2: VERIFICATION BY EYE‐TRACKING MEASUREMENT

We conducted an eye‐tracking experiment to examine whether the facial features computationally extracted in Study 1 were important for impression perception and how they relate to the perception of facial impressions and attention.

### Methods

We used 80 face images selected from the 290 male and 307 female images in the Chicago Face Database used in Study 1 for the stimuli, selecting 10 images for each race and sex, extracting them in order of proximity to the average age, considering the effect of age on impressions (He et al., 2021). We used these 80 facial images to generate 1200 facial images, creating 80 images for each of the three facial impressions (attractiveness, dominance, sexual dimorphism) and five levels of morphing (−3, −1.5, 0, 1.5 and 3 *SD*) using the model constructed in Study 1.

**FIGURE 3:**
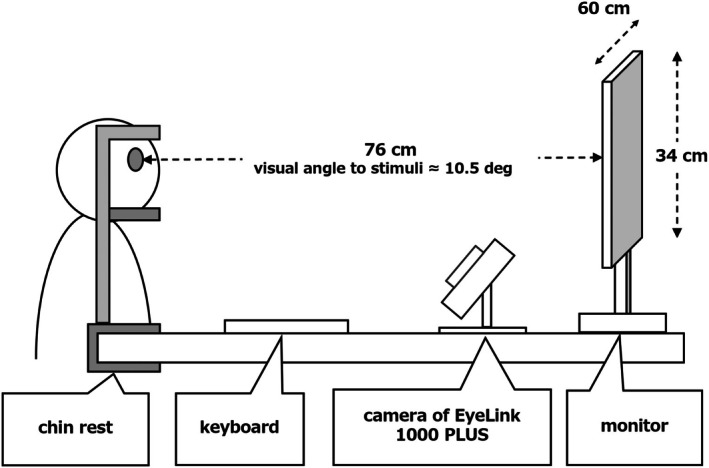
Experimental setup.

**FIGURE 4:**
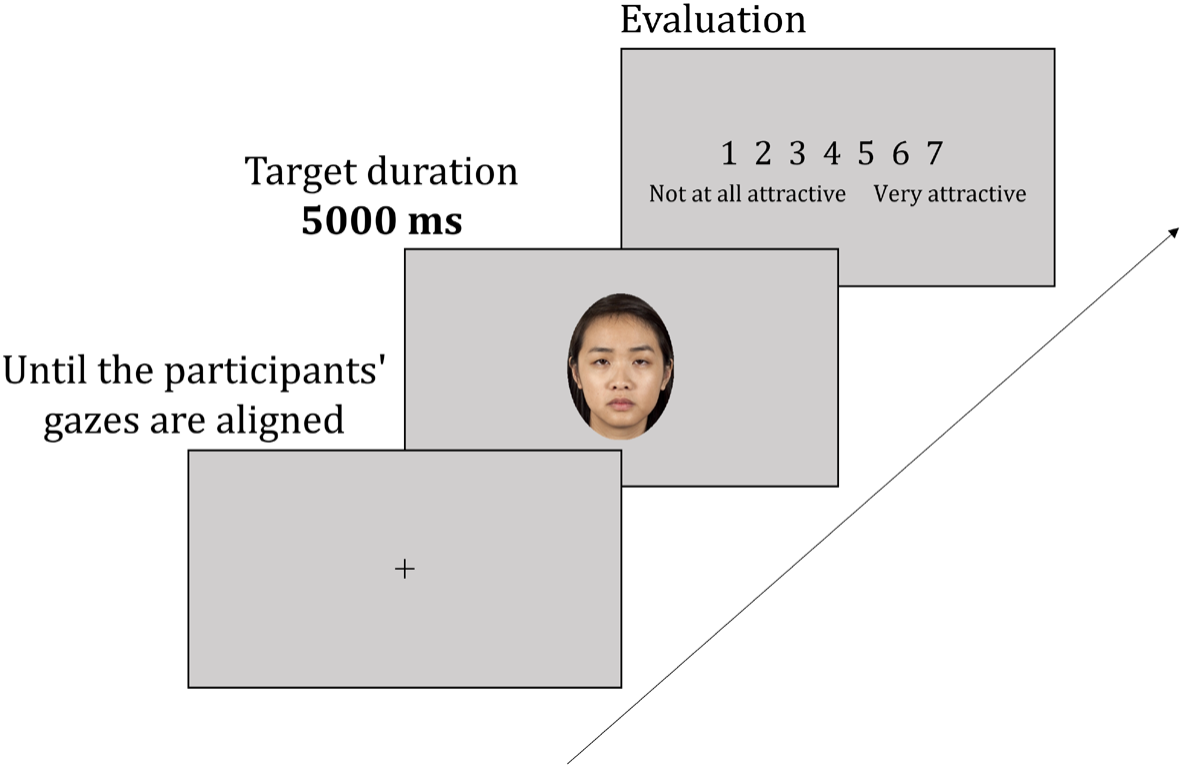
Example of the flow of one experiment trial.

The experiment involved 40 participants, comprising 20 men and 20 women, with an average age of 21.35 years (±1.75). Of these, 38 were Japanese and two were Chinese. This experiment was approved by an Ethics Committee in Japan, in accordance with the Declaration of Helsinki. Informed consent was obtained from the participants. Our analysis excluded data from five individuals who encountered issues, such as problems with contact lenses, glasses or malfunctions in the experimental setup, resulting in unsuccessful calibration. The final sample comprised 35 participants (16 men, 19 women; average age: 21.31 ± 1.86 years), of whom, 33 were Japanese and two were Chinese. Power analysis (*α* = .05; R package SIMR [Green & MacLeod, 2016]) confirmed that the sample size ensured at least an 80% power to detect the relation between morphological changes in morphing and impression scores. The effect size (*β*) for the morphological changes in morphing was set at 0.12 in accordance with a previous study (Sano & Kawabata, 2023). Participants were situated in individual rooms equipped with a chin rest, a monitor for visual stimuli and a keyboard for rating responses. The distance from their eyes to the monitor (ASUS VG278QR‐R) was standardized at approximately 76.0 cm (Figure 3). We used an EyeLink 1000 PLUS camera for eye‐tracking measurements. Participants evaluated the attractiveness, dominance and sexual dimorphism of facial images. Each trial in the rating tasks began with the presentation of a fixation point. Upon gaze fixation, the next screen appeared, displaying a facial image for 5000 ms. After the presentation of the facial image, the rating screen appeared. Participants evaluated the presented facial image on 7‐point scales for attractiveness (1 = *not at all attractive* to 7 = *very attractive*), dominance (1 = *not at all dominant* to 7 = *very dominant*) and sexual dimorphism (1 = *not at all masculine/feminine* to 7 = very masculine/feminine). The rating time was unlimited. After completing the rating, the participants proceeded to the next trial (Figure 4). Each impression rating task consisted of 80 trials. A total of 240 trials (80 × 3 blocks) were conducted. Participants could take breaks between blocks. The presentation order of the impression assessment task and face image was counterbalanced. Although 1200 image stimuli were prepared, each participant was assigned 240 trials, and the experiment was designed to cover all 1200 patterns with five participants. By using a large number of facial images, we aimed to verify universal effects that would not depend on the specific features of each facial image stimulus.

### Analysis 1: Linear mixed‐effects models for morphological changes and impression ratings

#### Methods

To examine the relation between manipulated facial features and obtained impression ratings, we conducted an analysis using linear mixed‐effects models (LMM). The analysis was performed using the lmer function in the lme4 package of R. We created three separate models for attractiveness, dominance and sexual dimorphism ratings. The models included fixed effects for morphological changes in facial stimuli, sex of facial stimuli, sex of participants and interactions of these factors. We specified random intercepts for participants and stimuli. Image and participant's sex were used as dummy variables: −0.5 for males and 0.5 for females.

#### Results

To provide an overview of the data, we reviewed the mean and SD of the impression scores recorded in the Chicago Face Database for the face images used and those obtained in this experiment. The Chicago Face Database was as follows: for male images, the mean attractiveness score was 3.07 (*SD* = 0.69), the mean dominance score was 3.20 (*SD* = 0.58) and the mean sexual dimorphism score was 4.70 (*SD* = 0.47); for female images, the mean attractiveness score was 3.54 (*SD* = 0.87), the mean dominance score was 2.72 (*SD* = 0.49) and the mean sexual dimorphism score was 4.60 (*SD* = 0.67). The experimental results were as follows: for male images, the mean attractiveness score was 3.50 (*SD* = 0.68), the mean dominance score was 3.74 (*SD* = 0.66) and the mean sexual dimorphism score was 4.57 (*SD* = 0.57); for female images, the mean attractiveness score was 3.46 (*SD* = 0.57), the mean dominance score was 3.90 (*SD* = 0.58) and the mean sexual dimorphism score was 4.39 (*SD* = 0.72).

In addition, we conducted a mixed analysis of variance (ANOVA), 2 image sex (male and female) × 4 image race (Asian, Black, Latin and White) × 2 data type (the dataset of this study and the Chicago database) design, to examine the effects on the evaluations of attractiveness, dominance and sexual dimorphism. The results are detailed in Figures S1–S3 in the Supplementary file. A difference was confirmed between the scores recorded in the dataset and those obtained in this experiment. This difference may be due to using face images deformed by geometric morphometrics in this study. Furthermore, some differences were also noted in the race scores obtained in this experiment. These may also be due to the differences between the evaluators in the Chicago Face Database and the Asian evaluators in this experiment.

To unify the model structure for attractiveness, dominance and sexual dimorphism impressions, we set each model to include morphological changes, image sex, participant sex and their interactions as fixed effects. We considered participants as random intercept effects to account for individual differences.

For all three impressions, the LMM analysis revealed significant effects for morphological changes (for attractiveness, dominance and sexual dimorphism respectively: estimate [Est] = 0.101, 0.171, 0.115; standard error [*SE*] = 0.011, 0.013, 0.012; *t* = 9.213, 13.617, 9.327, *p* < .001). For attractiveness, significant effects were also found for the interaction between participant and image sex (Est = 0.227, *SE* = 0.093, *t* = 2.439, *p* < .005). For dominance, the LMM analysis also showed significant effects for image sex (Est = 0.137, *SE* = 0.053, *t* = 2.580, *p* < .01), interaction between morphological changes and image sex (Est = 0.061, *SE* = 0.025, *t* = 2.450, *p* < .05) and interaction between participant sex and image sex (Est = 0.415, *SE* = 0.106, *t* = 3.895, *p* < .001). For sexual dimorphism, the LMM analysis demonstrated significant effects for image sex (Est = −0.195, *SE* = 0.052, *t* = −3.719, *p* < .001), interaction between participant and image sex (Est = 0.303, *SE* = 0.105, *t* = 2.890, *p* < .005) and interaction between morphological changes, participant and image sex (Est = −0.100, *SE* = 0.049, *t* = −2.027, *p* < .05). Other main effects and interactions were not significant.

**FIGURE 5:**
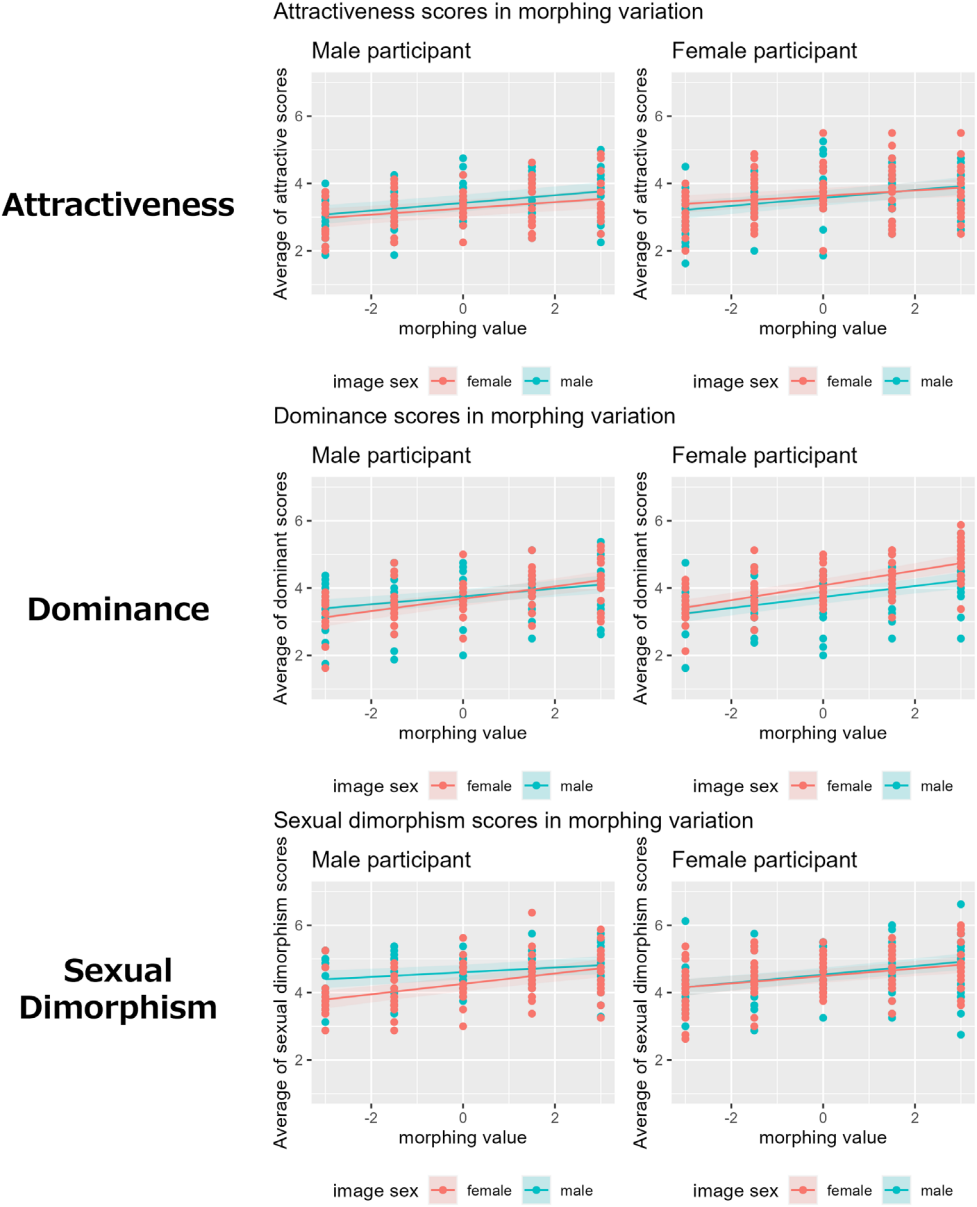
Results of linear mixed‐effects models for attractiveness, dominance and sexuality. The thin widths of the regression lines indicate 95% confidence intervals.

These results indicated that the manipulated facial features correspond to actual impression perception (Figure 5). For dominance impressions, the interaction with image sex suggests that the effect of morphological changes was more pronounced for female images. For sexual dimorphism impressions, the interaction between participant and image sex indicate that the effect of morphological changes was particularly significant when male participants evaluated female images. Thus, morphometric manipulations corresponded to impression evaluations, and evaluation tendencies regarding morphological changes varied based on participant and image sex.

### Analysis 2: Visualization of fixation area heatmaps

#### Methods

To examine which facial regions the participants focused on during each impression evaluation, we created heatmaps using the fixation map creation function of EyeLink. The analysis focused on the 5000 ms duration when facial images were presented. Heatmaps were generated for each combination of three impressions (attractiveness, dominance, sexual dimorphism), two image sexes (male, female), two rater sexes (male, female) and five levels of morphological changes (−3, −1.5, 0, 1.5, 3 *SD*), for a total of 60 visualized patterns.

#### Results

**FIGURE 6:**
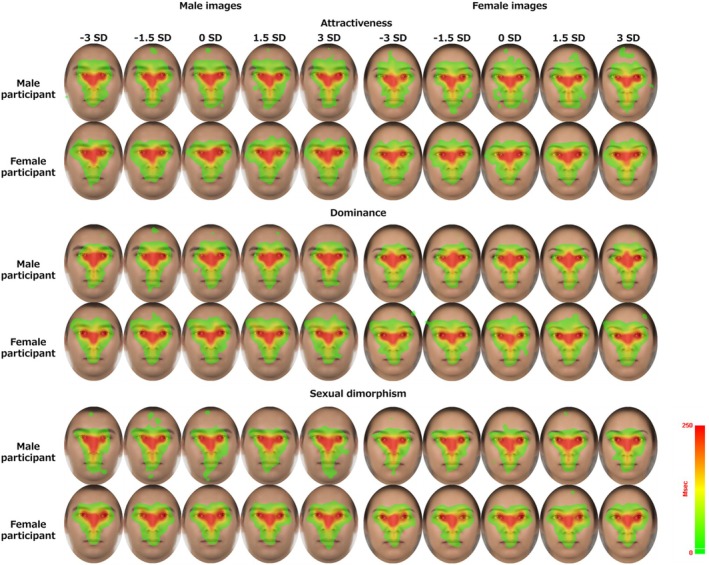
Heatmap visualization results of the fixation area during the presentation of face images. The red colour indicates a longer fixation time.

During each impression evaluation, the participants consistently focused on the eye, nose and mouth regions (Figure 6). This result aligns with previous studies of eye‐tracking on facial images (Barton et al., 2006; Kwart et al., 2012; Walker‐Smith et al., 1977). However, under various morphing conditions, image sex and participant sex, detailed differences in fixation areas could not be discerned solely from this result. To investigate which facial regions statistically differed across conditions, we conducted pixel‐wise LMM analyses.

### Analysis 3: Statistical analysis of fixation areas using pixel‐wise LMM

#### Methods

To investigate the detailed differences in fixation areas based on experimental conditions, we conducted a pixel‐wise LMM analysis using *i*Map4 (Lao et al., 2017). This analysis can consider all regions at the pixel‐level and visualize statistically significant differences in fixation duration based on condition. We used the filter parameters built into EyeLink (saccade velocity threshold = 30°/s, saccade acceleration threshold = 8000°/s^2^) and its built‐in algorithm to parse the images into fixations and saccades automatically. We defined blinks as intervals where two measured features of the eye image (pupil centre and corneal reflex) were missing. The remaining interval was defined as fixations. We used only the fixations in the analysis, which focused on the 5000‐ms duration when facial images were presented. The independent variables in the model included the morphological changes of facial stimuli as fixed effects and participants as random intercepts. We used three categorical variables to examine the differences between the morphing conditions in the analysis: positive (+1.5, +3 *SD*), negative (−1.5, −3 *SD*) and 0 *SD* conditions. Each variable was standardized. Considering the differences in morphological features by image sex, we created separate models for each image sex. Additionally, we conducted the analysis separately for each facial impression. Subsequently, we calculated the differences by subtracting conditions on the positive side of morphing from conditions on the negative side for morphing conditions. For participant sex conditions, differences were calculated by subtracting the female participant side from the male participant side. We applied the two‐tailed pixel test (*p* < .05) to the difference fixation maps. We also applied a false discovery rate to account for Type I errors due to multiple comparisons.

#### Results

**FIGURE 7:**
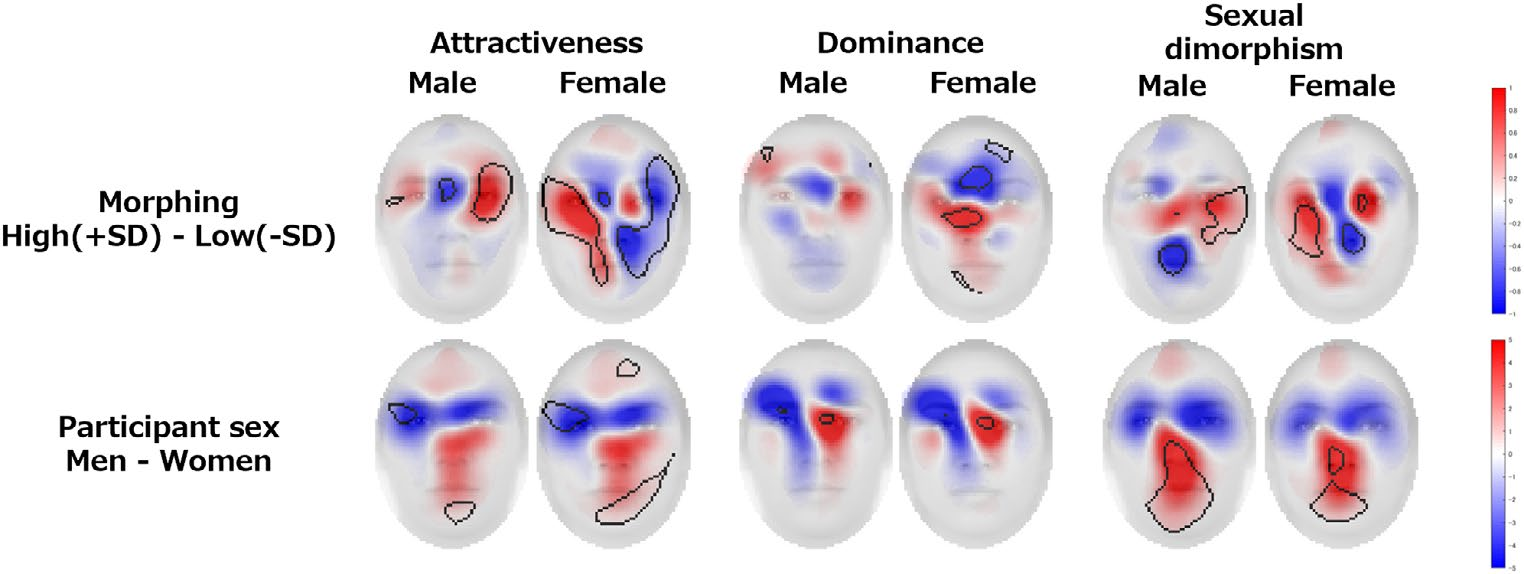
Pixel‐wise LMM results during presentation of face images. The upper panel shows the difference in results from the +*SD* side to the −*SD* side of the morphing condition. The redder the heatmap, the higher the *β* value on the +*SD* side, and the bluer, the lower the *β* value on the −*SD* side. Similarly, the lower panel shows the difference in results from male to female participants in the participant sex condition. The redder the heatmap, the higher the *β* value for males, and the bluer, the lower the *β* value for females. The area surrounded by the black line indicates the significant region.

The results of the pixel‐wise LMM are shown in Figure 7. Differences were identified by each impression, image sex and participant sex. In the attractiveness evaluation task for male images, the participants tended to focus more on the eye region when morphing was in the positive direction and the region between the eyebrows when morphing was in the negative direction. For female images, positive morphing conditions led to increased focus on the right cheek region, whereas negative morphing conditions resulted in attention to the left eye and nose regions. Regarding participant sex conditions, male participants tended to focus on the nose and mouth, whereas female participants concentrated on the areas around the eyes and eyebrows, regardless of image sex. Positive heat maps extended to the nose and mouth, with areas around the mouth being particularly significant.

In the dominance evaluation task, for male images, positive morphing conditions led participants to focus on the left eye and forehead regions, whereas negative morphing conditions directed attention to the right cheek, mouth, and between the eyebrows regions. For female images, positive morphing conditions resulted in increased attention to the nose region, whereas negative morphing conditions led to attention on the forehead and between the eyebrows. Concerning participant sex conditions, male participants tended to focus on the nose and the region between the eyebrows; female participants concentrated on the areas around the right eyes and eyebrows regardless of image sex.

In the sexual dimorphism evaluation task, for male images, positive morphing conditions led participants to focus on the areas around the left eyes and nose, whereas negative morphing conditions directed attention to the eyebrow and mouth regions. For female images, positive morphing conditions increased attention to the right cheek and left eye regions, whereas negative morphing conditions resulted in focus on the nose and the region between the eyebrows. Regarding participant sex conditions, male participants tended to focus on the nose and mouth regions, while female participants concentrated on the areas around the eyes and eyebrows.

Overall, we found that when observing stimuli with different morphological features, there were differences in attention to the eyes, eyebrows and the area between the eyebrows. In the attractiveness and sexual dimorphism evaluations, we also found that male participants tended to pay more attention to the nose and mouth area than female participants. In comparison, female participants tended to pay more attention to the eye area.

### Discussion

In Analysis 1 using LMM, we observed a correspondence between the degree of deformation of morphological features manipulated through geometric morphometrics and the perceived facial impressions.

In Analysis 2, as in the literature (Barton et al., 2006; Kwart et al., 2012; Walker‐Smith et al., 1977), fixation was found around the eyes, nose and mouth in all conditions, and the details of the differences between the different evaluation tasks could not be confirmed. However, Analysis 3 suggested that the facial features used as cues may differ depending on the evaluation task. This is consistent with the results of studies that refer to different regions depending on the evaluation task (Calvo et al., 2019). However, given that the features emphasized in the face images in this study differed by task, further research is needed to determine the details.

Regarding Analysis 3, the results suggested that, in addition to the areas around the eyes, the nose and cheeks may play a crucial role in attractiveness judgements, especially for female images. Considering the reported importance of the nose, eyes and mouth in attractiveness judgements (Terry & Davis, 1976), our participants may have focused on the nose and eyes regions. Moreover, male participants tended to focus on the nose and mouth, whereas female participants focused on the eyes and eyebrows. This aligns with findings that, in facial expression recognition, women tend to focus on the eyes, whereas men focus more on the nose and mouth (Vassallo et al., 2009).

Moreover, the eyes, eyebrows, between the eyebrows and nose served as cues for dominance judgements. Previous research has shown that increased emphasis on dominance impressions is associated with raised eyebrows and facial structures resembling expressions of anger (Montepare & Dobish, 2003). Additionally, the width of the nose has been linked to perceptions of dominance (Kleider‐Offutt et al., 2021), supporting our finding that participants relied on cues from the regions around the eyes and nose to judge dominance.

In the sexual dimorphism evaluation, the eyes, eyebrows, nose and cheeks served as cues. Sexual dimorphism in faces is associated with raised eyebrows in males, attributed to testosterone and larger eyes in females, attributed to oestrogen (Little et al., 2011; Rhodes, 2006; Thornhill & Gangestad, 1999). The observed focus on these facial regions implies the importance of these features in judgements of sexual dimorphism. Additionally, we noted differences in fixation patterns in the mouth region based on participant sex, consistent with the reported tendency of women to focus more on the eyes and men on the nose and mouth (Vassallo et al., 2009).

## STUDY 3: ANALYSIS USING DEEP LEARNING METHODS

Using the face images generated in Study 1, we analysed the facial features deemed important for each impression using deep learning methods. We then compared them with the results of the fixation areas obtained from the eye‐tracking experiment in Study 2. We examined the similarities and differences between the areas perceivers fixated on when evaluating facial images and the areas that the deep learning model used as predictors for each impression.

### Analysis 1: Accuracy verification of deep learning models

#### Methods

According to the model constructed in Study 1, we used 290 male and 307 female face images to prepare stimuli and generated 1450 male and 1535 female images with five levels of manipulation of the face shape involved in each impression (attractiveness, dominance and sexual dimorphism) to expand the data for learning. We assigned labels based on the impression scores of the original images. As in Study 1, we clipped each face image into an oval shape to remove the influence of the background. These face images were used as training data for the deep learning model. We used tensorflow/keras (Version.2.6.0) for the analysis.

**FIGURE 8:**
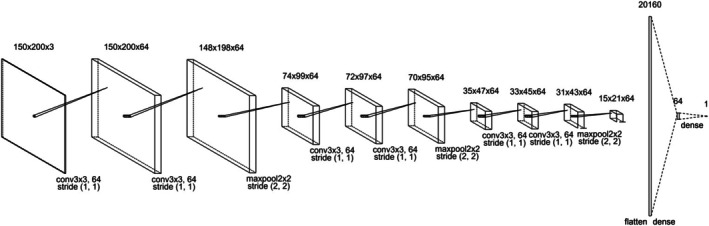
Architecture of CNN model: The numbers in the upper row indicate each layer's image and size. The values in the lower row indicate the convolution and max pooling layer values and stride values.

**FIGURE 9:**
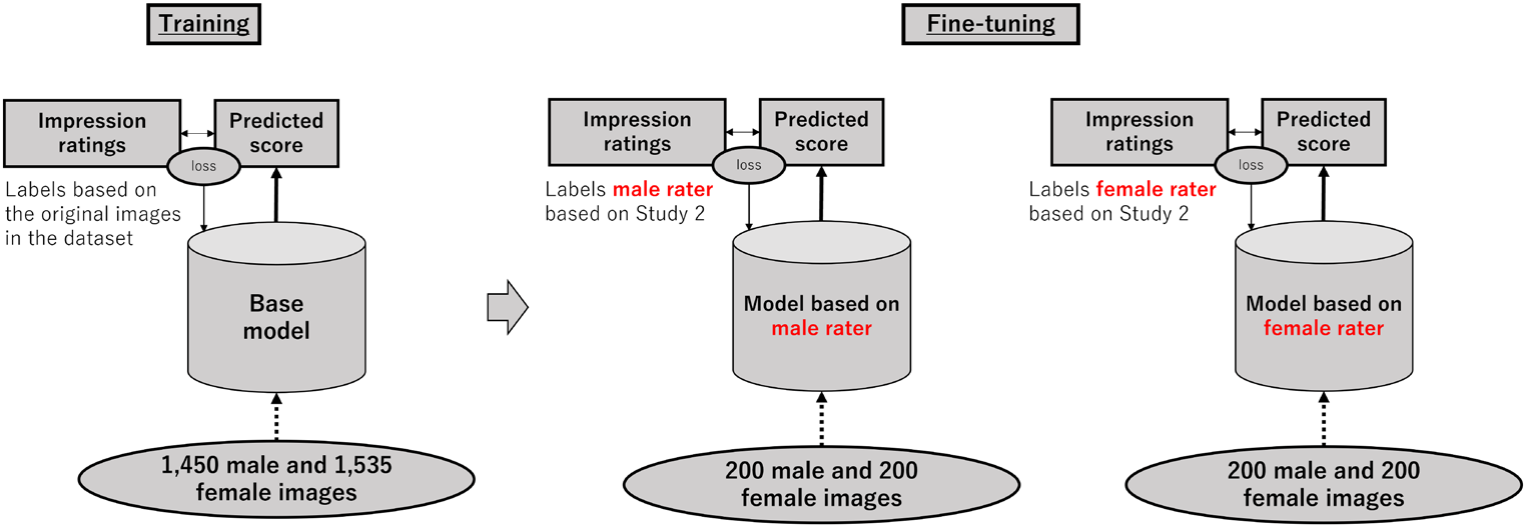
Overview of CNN model construction and fine‐tuning methods.

Using these face images and labels, we constructed a CNN model using the original image data by deep learning methods. Considering the difference in results depending on the image sex, we created two models, for learning male and female images. We employed a six‐layer CNN model, with a batch normalization layer (Ioffe & Szegedy, 2015) placed immediately after the CNN layer and a max pooling layer placed at every second CNN layer. The activation function was Relu, and the final layer was linear. The optimization function was Adam, and the batch size was fixed at 16 with a learning rate of 0.001. Training was performed in 300 epochs. The model structure and parameters were designed based on a previous study (Sano & Kawabata, 2023). We set the input size considering computational resources and the trends of morphological changes. The architecture of the model is shown in Figure 8. Considering the differences in the results between raters, the ratings of the 200 male and 200 female images obtained in Study 2 were divided into two groups: based on rater sex and each group was used as label data to perform fine‐tuning on the base model. The fine‐tuning technique used for this base model enabled learning even with as few as 200 images. We set each parameter to the same value for this fine‐tuning. Training was again performed for 300 epochs. To confirm the model's reliability, we used a five‐part cross‐validation method to verify the model's accuracy. Based on previous studies (Sano & Kawabata, 2023; Xu et al., 2017), we used the Pearson correlation between predictions and correct labels as the accuracy measure. We also constructed a model trained on all data to extract important regions; we confirmed its accuracy when all data were predicted. Models were constructed for each impression, sex of the rater and image sex. For the one male image that was not assigned to the sexual dimorphism impression score owing to the exclusion of participant data in Experiment 2, we used the mean value of the sexual dimorphism score of the male image to supplement the impression score. The constructed model and fine‐tuning methods are shown in Figure 9.

#### Results

Table 1 shows the accuracy scores of the attractiveness, dominance and sexual dimorphism models. The results confirmed that the prediction accuracy differed depending on the rater. Therefore, the features used as predictors in the deep learning model may also differ depending on the rater. Given the low data volume for the test and training, at 200 images each, we observed differences in accuracy depending on the data validation. Indeed, the generalization performance of the models could have been higher. However, we aimed not to construct a model with high generalizability but to interpret important facial features using the obtained evaluation labels. Therefore, we confirmed the accuracy of the model when all data were trained, and 200 labels were predicted for each.

**TABLE 1:** Results of accuracy of five‐part cross‐validation using Pearson correlation as the criterion.

Model	Rater	Image sex	Test 1	Test 2	Test 3	Test 4	Test 5	Average
Attractiveness	Male	Male	0.253 n.s.	0.417 **	0.61 ***	0.602 ***	0.491 **	0.474
Attractiveness	Male	Female	0.586 ***	0.527 ***	0.333 *	0.404 **	0.319 *	0.434
Attractiveness	Female	Male	0.681 ***	0.703 ***	0.534 ***	0.709 ***	0.762 ***	0.678
Attractiveness	Female	Female	0.402 *	0.585 ***	0.557 ***	0.365 *	0.52 ***	0.486
Dominance	Male	Male	0.528 ***	0.352 *	0.476 **	0.409 **	0.510 ***	0.455
Dominance	Male	Female	0.590 ***	0.686 ***	0.541 ***	0.348 *	0.492 **	0.532
Dominance	Female	Male	0.405 **	0.591 ***	0.635 ***	0.482 **	0.577 ***	0.538
Dominance	Female	Female	0.517 ***	0.506 ***	0.601 ***	0.605 ***	0.692 ***	0.584
Sexual dimorphism	Male	Male	0.421 **	0.452 **	0.522 ***	0.578 ***	0.306 n.s.	0.456
Sexual dimorphism	Male	Female	0.471 **	0.492 **	0.686 ***	0.473 **	0.702 ***	0.565
Sexual dimorphism	Female	Male	0.192 n.s.	0.354 *	0.437 **	0.665 ***	0.582 ***	0.446
Sexual dimorphism	Female	Female	0.616 ***	0.677 ***	0.546 ***	0.607 ***	0.642 ***	0.618

*Note*: Rater indicates the sex of the rater of images used for training and prediction. Image sex indicates the sex of the image set used for training and prediction. Tests 1–5 indicate the results of each cross‐validation test. The values represent Pearson correlation values, and the average indicates their average values.

* *p* < .05.

** *p* < .01.

*** *p* < .001.

Table 2 shows the results for the accuracy of the models when all data were trained. In line with our aim, we extracted important facial features using this model, which had high prediction accuracy in the range of the training data.

**TABLE 2:** Results of accuracy for all data using Pearson correlation as the criterion.

Model	Rater	Image sex	Correlation
Attractiveness	Male	Male	0.992***
Attractiveness	Male	Female	0.988***
Attractiveness	Female	Male	0.995***
Attractiveness	Female	Female	0.994***
Dominance	Male	Male	0.985***
Dominance	Male	Female	0.980***
Dominance	Female	Male	0.989***
Dominance	Female	Female	0.983***
Sexual dimorphism	Male	Male	0.972***
Sexual dimorphism	Male	Female	0.994***
Sexual dimorphism	Female	Male	0.986***
Sexual dimorphism	Female	Female	0.991***

*Note*: Rater indicates the sex of the rater of images used for training and prediction. Image sex indicates the sex of the image set used for training and prediction. The Pearson correlation is shown when all 200 male images or all 200 female images are trained and these are used as test data.

* *p* < .05.

** *p* < .01.

*** *p* < .001.

### Analysis 2: Visualization results with grad‐CAM

#### Methods

To confirm the details of the face regions of interest for the deep learning model, we used the constructed model to visualize the hidden layers by Grad‐CAM (Selvaraju et al., 2017), which is excellent for this purpose. The features important for predicting facial attractiveness were extracted by averaging over the 200 male and 200 female images treated in Study 2 for each facial shape varied in five levels. Models were constructed for each impression, and a total of 60 visualization results were calculated for three impressions (attractiveness, dominance and sexual dimorphism) × two image sex (male and female) × two rater sex (male and female) × five shape changes (−3, −1.5, 0, 1.5, 3 *SD*).

#### Results

**FIGURE 10:**
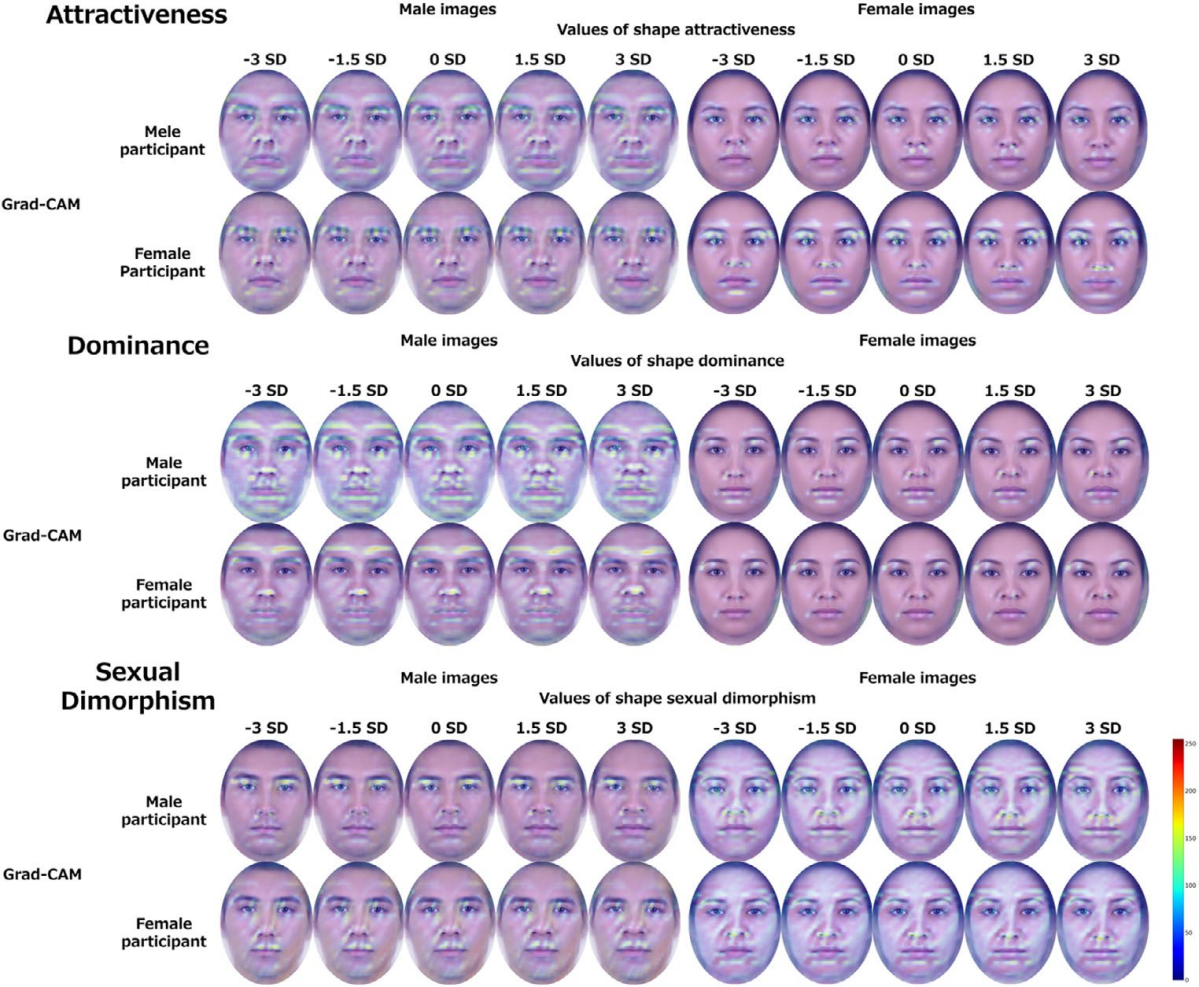
Grad‐CAM visualization results. The brighter areas of the heatmap indicate that each impression was more important in predicting the results. The heatmaps from 0 to 255 were superimposed on each image.

We confirmed the Grad‐CAM heatmap for regions with values significantly greater than 0 by applying a false discovery rate that accounts for type I error due to multiple comparisons to a per‐pixel *t*‐test (*p* < .05) (See Figures S4–S6). Each impression, image sex and rater sex identified differences. The visualization results are shown in Figure 10.

The attractiveness analysis revealed that for male images, the deep learning model based on male participants' ratings had stronger heatmaps around the eyes, eyebrows, superciliary arches, nose, mouth and chin. Female participants' ratings also highlighted the eyes, brow area, nose, mouth and chin. For female images, male participants' heatmaps were stronger around the eyes, eyebrows, under the nose and mouth, while female participants emphasized the eyes, eyebrows and nose tip. Despite minor differences between morphing conditions, these areas remained critical for prediction.

In the dominance analysis, male images showed stronger heatmaps around the eyes, eyebrows, superciliary arches, nose and mouth for both male and female participants, with a notable emphasis on superciliary arches for women. For female images, male participants' heatmaps were stronger around the eyebrows, mouth and chin, while female participants focused on the outer corners of the eyes, superciliary arches and mouth. These areas were consistently important across morphing conditions.

The sexual dimorphism analysis indicated that male images had stronger heatmaps around the eyes, nose and mouth for male participants and around the eyes, eyebrows, nose and mouth for female participants. For female images, male participants' heatmaps covered the eyes, eyebrows, superciliary arches, nose, chin and facial skin, whereas female participants' heatmaps included the eyes, eyebrows, superciliary arches, nose, mouth and facial skin. While morphing condition differences were not significant, these areas were essential across all conditions.

These results showed that in addition to the eyes, nose and mouth, broader areas such as the eyebrows, superciliary arches and chin were important in predicting each impression.

**FIGURE 11:**
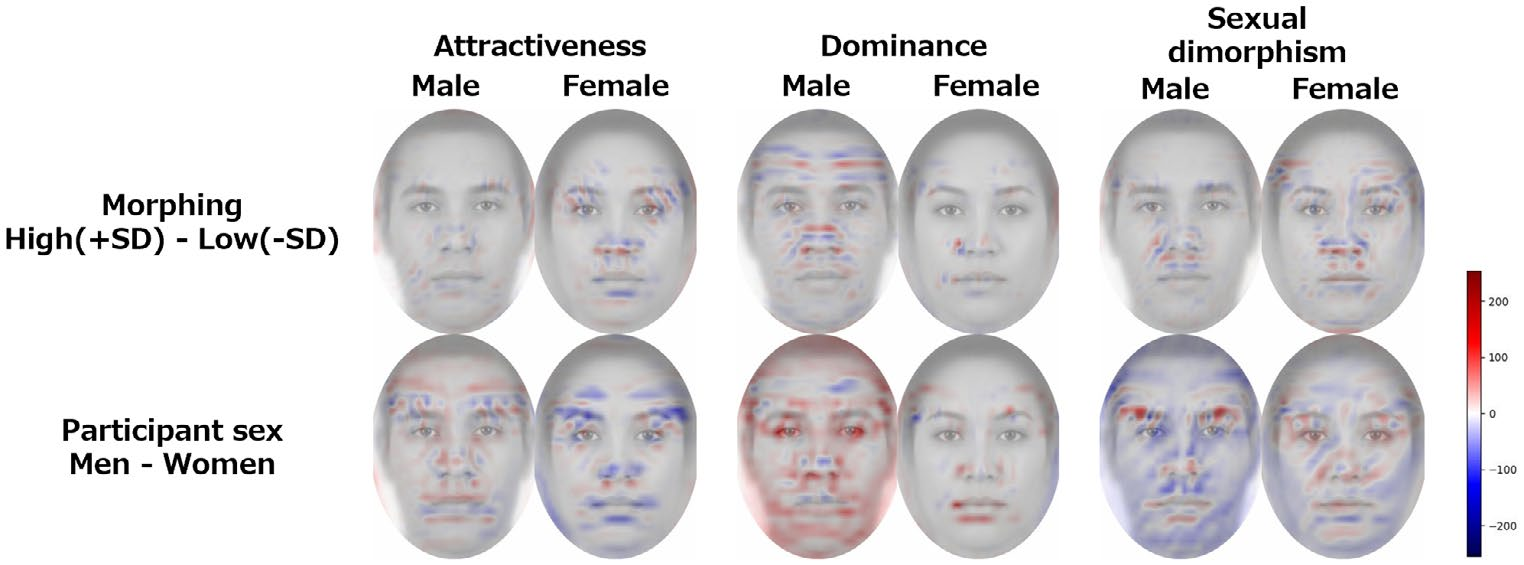
Grad‐CAM results of the differences by facial morphometric change and by rater sex. The upper panel shows the difference in results from the +*SD* side to the −*SD* side of the morphing condition, where the redder the heatmap, the higher the heatmap value on the +*SD* side, and the bluer the lower the heatmap value on the −*SD* side. Similarly, the lower panel shows the difference in results from male to female participants in the participant sex condition, where the redder the heatmap, the higher the heatmap value for males and the bluer the lower the heatmap value for females.

To further investigate the differences by facial morphometric change and rater sex, the differences between the positive (+1.5, +3 *SD*) and negative (−1.5, −3 *SD*) conditions and the heatmap results for the male and female rater conditions were calculated and visualized, focusing on the face areas clipped in ellipses (Figure 11). In the morphing condition, we found some differences in features such as altered noses and eyebrows. In the sex condition of the raters, there were differences in the eyebrow and superciliary arches areas for attractiveness, in the overall area such as facial contour for dominance, especially in the male face, and in the overall area and eyebrow area for sexual dimorphism.

### Analysis 3: Distance calculation between the distributions of heatmap values for fixation duration and grad‐CAM

#### Methods

We analysed the Kullback–Leibler divergence (KLD) between the distributions of the fixation time heatmaps extracted in Study 2 and the heatmap values extracted by Grad‐CAM to investigate whether the eye‐tracking experiment and Grad‐CAM measured the same variables. The closer the value of KLD to 0, the more approximate the two distributions are. The fixation time heatmap and the Grad‐CAM heatmap of each face image were normalized as a probability distribution whose sum is 1, and means were calculated for each of the impression, rater and image sex conditions.

Furthermore, we performed a correlation analysis on the heatmap values extracted by Grad‐CAM and the area of interest (AOI) at gaze time extracted in Study 2 to determine whether the eye‐tracking experiment and Grad‐CAM measured the same variables.

#### Results

The results are shown in Table 3. Given that the model performance that produces highly accurate saliency maps as metrics has a KLD below 1 (e.g. Kummerer et al., 2017), the distribution of fixation time and Grad‐CAM can be interpreted as being very far apart.

**TABLE 3:** Results of KLD of the distributions of heatmaps between fixation time and Grad‐CAM.

Model	Rater	Image sex	KLD
Attractiveness	Male	Male	14.31
Attractiveness	Male	Female	20.85
Attractiveness	Female	Male	16.20
Attractiveness	Female	Female	15.57
Dominance	Male	Male	10.62
Dominance	Male	Female	20.93
Dominance	Female	Male	17.09
Dominance	Female	Female	22.54
Sexual dimorphism	Male	Male	18.35
Sexual dimorphism	Male	Female	12.05
Sexual dimorphism	Female	Male	13.95
Sexual dimorphism	Female	Female	9.85

The results of the correlation analysis showed no significant correlations (See Figures S7–S10).

### Discussion

The Grad‐CAM analysis of attractiveness impressions showed stronger heatmaps around the eyes, eyebrows, under the nose and around the chin for male images based on male participants' ratings. Female participants' results were similar and consistent with previous studies (Sano, 2022, 2023; Sano & Kawabata, 2023). For female images, male participants focused on the eyes, eyebrows, under the nose and around the mouth, whereas female participants emphasized the eyes and tip of the nose. Previous studies noted skin regions as important in female images (Sano, 2022, 2023; Sano & Kawabata, 2023). This study suggests facial morphology is a strong attractiveness predictor based on training on images with deformed features.

For dominance impressions, male participants' ratings showed stronger heatmaps for the eyes, eyebrows, superciliary arches and nose and mouth areas for male images. Female participants had similar results, highlighting the superciliary arches. These findings align with studies linking raised eyes and eyebrows to dominance (Montepare & Dobish, 2003; Třebický et al., 2013). For female images, male participants emphasized the eyebrows, while female participants focused on the eyes and mouth.

For sexual dimorphism impressions, male participants' ratings of male images showed stronger heatmaps around the eyes, eyebrows, under the nose and mouth. Female participants showed similar results. Sexual dimorphism is crucial for attractiveness (Little et al., 2011; Rhodes, 2006; Thornhill & Gangestad, 1999). For female images, heatmaps around the eyes, eyebrows, nose and mouth were stronger for both groups, with skin areas also indicated. The contrast between eyes, lips and skin influences sex recognition (Russell, 2003). Skin areas were extracted in addition to morphological features. Featured of facial attractiveness and sexual dimorphism are separable (Nakamura & Watanabe, 2020), with distinct jaw and chin forms contributing to each (Valenzano et al., 2006). Deep learning models may extract distinct feature regions.

However, Analyses 3 showed no heatmap similarity or correlation between Grad‐CAM and fixation time. In the eye‐tracking experiment, fixation time serves as the measure, while in the deep learning method, the contribution of features to the predictions of the model serves as the measure. The results suggest a discrepancy in the interpretation of feature importance between the two approaches. It appears that the facial features computationally corresponding to the facial impression scores do not necessarily correspond to the areas visually attended to. Despite little correlation between Grad‐CAM and fixation time, there was a comparative difference in heatmap values across image sex on the Grad‐CAM axis, suggesting that the importance of the eyes, nose and mouth in predicting scores may differ by image sex.

## GENERAL DISCUSSION

In this study, we used eye tracking and deep learning to identify facial features important for facial impressions using face images altered with geometric morphometrics. The results consistently supported previous research in each study. However, by calculating KLD, Analysis 3 of Study 3 revealed that the heatmap values obtained by Grad‐CAM were not similarly distributed as the fixation map values. Moreover, little significant correlation was found in the AOI. Essentially, facial features that correspond computationally to the facial impression scores may not correspond to visually attended areas. Deep learning models are optimized to predict attractiveness, dominance or sexual dimorphism scores, extracting information about regions where image differences dominate.

Visual attention is guided by both the features of the face image and the evaluation task, as the area of attention does not necessarily coincide with the key features that influence impression formation. Indeed, there are both stimulus‐driven and goal‐driven eye movement strategies during facial emotion recognition (Schurgin et al., 2014), and visual saliency of images is considered a feature that attracts visual attention (Itti & Koch, 2000). Conversely, Grad‐CAM visualizes the regions where deep learning models contribute to predictions and is based on the patterns learned by the models. The model automatically extracts facial features from large amounts of data and predicts impressions based on them. Therefore, the areas of interest to the model depend not on visual prominence but on the patterns of facial features that the model learns. Therefore, the discrepancy in the results may stem from human visual attention being based on visual saliency, and the patterns extracted by the model in a data‐driven manner are different. However, for example, Grad‐CAM specifically extracted the eyebrow and superciliary arches regions when predicting the dominant impression of male faces. Although these may be areas that do not receive much visual attention, eyebrows are related to the perception of angry facial expressions and are considered one of the most important factors in determining the impression of dominance (Montepare & Dobish, 2003). In addition, male faces have more prominent features related to dominance, such as a more pronounced chin, wider forehead and cheekbone structure (Little et al., 2011; Rhodes, 2006; Thornhill & Gangestad, 1999), and it is possible that the model detected and emphasized these more extensively than with female faces, especially in the case of dominance impressions. Thus, visualization of the hidden layers of deep learning models by Grad‐CAM may contribute to extracting the determinants of facial impressions independent of visual attention.

In addition, despite differences in the overall trends between eye tracking and deep learning, the results of Studies 1, 2 and 3 support the findings of several previous studies, and some features were consistent. For attractiveness, the results indicated large eyes, sharply angled contours and elevated eyebrows, as revealed by geometric morphometrics, and that the eye and nose areas were used as cues in evaluating the impression of attractiveness, as revealed by the eye‐tracking experiment. The regions around the eyes and nose were also found to be important by the deep learning model. Indeed, the eyes and nose are critical features in attractive faces (Terry & Davis, 1976), as also reported by studies using geometric morphometrics around the eye area (Nakamura et al., 2020; Windhager et al., 2011, 2018), eye‐tracking experiments (Kwart et al., 2012) and deep learning models (Sano, 2022, 2023; Sano & Kawabata, 2023). Nasal regions have also been reported to be important in studies using geometric morphometrics and eye‐tracking experiments (Windhager et al., 2011, 2018; Zhang et al., 2017). Meanwhile, previous studies using deep learning models have not extracted the nose region (Sano & Kawabata, 2023). Our results may be attributed to the model being trained on face images generated by a geometric morphometric method. Thus, despite differences in the degree of features extracted, morphological features that are the focus of both eye tracking and deep learning may be critical features in facial impressions.

Geometric morphometrics further revealed that the dominant face tended to have a particularly elevated brow, and analysis of differences in the morphing conditions of the fixation area showed that the brow and nose area were used as cues in evaluating the dominance of the face. Furthermore, we found that the eye, eyebrow and superciliary arches regions were important through the deep learning model. The more the dominance impression is emphasized, the more the eyes and eyebrows are lifted, resembles the facial structure during the expression of anger (Montepare & Dobish, 2003). Notably, a male face with a raised superciliary arches gives a masculine and aggressive impression (Třebický et al., 2013). Our results confirmed these earlier findings.

For sexual dimorphism, the geometric morphometrics revealed that the male face tended to emphasize the nose and mouth features, whereas the female face tended to have more rounded facial features. In the case of female images, the cheek area was found to be a cue for sexual dimorphism. In addition, we found that the area around the eyes was also extracted by the deep learning model and that the skin area was also important in the case of female images. Additionally, we found that the skin areas, such as cheeks, were important in the femininity of female images. In previous reports, these features are related to masculinity and femininity by testosterone in men and oestrogen in women (Little et al., 2011; Rhodes, 2006; Thornhill & Gangestad, 1999). Moreover, the luminance contrast between the eyes/lips and surrounding skin of a female face affects sex recognition more than that of a male face (Russell, 2003). Our results also showed that in addition to morphological features, skin regions were also considered and extracted by the deep learning model.

In each impression evaluation, differences by participant sex in fixation at facial parts were observed. In addition, the deep learning extraction results confirmed a difference by sex of the rater that differed from that in the eye‐tracking results. In the eye‐tracking experiments, male subjects tended to pay more attention to the nose and mouth than female participants, and female participants tended to pay more attention to the eye area, especially in the evaluation of attractiveness and sexual dimorphism. On the other hand, the deep learning analysis also revealed a wide range of differences in important facial features and their ratings by the sex of the rater. In particular, there were differences in the importance of eyebrows, superciliary arches, skin and overall contour, which varied by image sex. Thus, just as it is known that attractiveness ratings differ between same‐ and opposite‐sex raters (Little et al., 2001, 2002), trends in factors of facial impression factors that do not depend on visual attention may be different.

This study applied a simple CNN model and a typical visualization method, Grad‐CAM, to explore facial features important for impressions. Although the obtained results differed that of from eye tracking, developing a model produces results similar to fixation maps is technically feasible by applying an advanced deep learning model. For example, it is possible to develop a model close to human visual attention by making the results of eye tracking itself the training target of the model or by considering the saliency of image features (e.g. Kummerer et al., 2017). With progress, models can be used to perform simulation analysis of where people direct their attention when evaluating facial impressions without eye‐tracking experiments. On the other hand, using models that do not rely on visual attention, as in our study, would contribute to the search for hidden and essential facial features. These technological developments in AI should provide new perspectives for future facial impression research.

In this study, the participants were Asian, comprising Japanese and Chinese individuals. Therefore, different effects may have occurred in the evaluation of same and different‐race images. In fact, in the results using Grad‐CAM, heatmaps tended to scatter over the entire skin area, not just facial morphological features, especially for dominance in male images and sexual dimorphism in female images. This suggests that Asians may have used skin colour as a cue in their impression ratings (Lu et al., 2021); although the Chicago Face Database scores are the average of ratings by raters of various races, future studies are expected to take into account differences based on the rater's race.

This study utilized the Chicago Face Database, which includes scores for attractiveness, dominance and sexual dimorphism. It specifically included images of Asians, considering that the participants were Japanese and Chinese. While this dataset is superior in that it is well‐controlled for facial images, allowing for the exclusion of background, photographic appearance, facial expressions and other factors not included in this study, it has drawbacks that raise concerns about real world validity (Jenkins et al., 2011; Sutherland et al., 2013). In a previous study using geometric morphometrics and deep learning on the SCUTFBP5500 dataset (Liang et al., 2018), which is not strictly controlled, regions such as the eyes, surroundings and eyebrows were extracted as features related to facial attractiveness (Sano & Kawabata, 2023). Although the same regions were extracted in our study, some differences were observed in detailed regions such as the skin; therefore, the results may depend on the set of facial images used. However, SCUTFBP5500 does not include impression scores other than attractiveness, and the 10 k US Adult Faces Database (Bainbridge et al., 2013), which contains a variety of impression scores, has limitations, such as the limited number of races included. Therefore, it is imperative to construct a dataset containing diverse facial images and impression scores in the future.

This study used geometric morphometrics to define facial morphological features broadly. Additionally, deep learning methods were employed to handle information on the entire image, including skin and other information that cannot be considered by geometric morphometrics alone. In a previous study, machine learning was used to calculate critical facial features that predict impressions of reliability and dominance (Jaeger & Jones, 2022). While machine learning methods are helpful in confirming the correspondence between designed features and predicted impression scores, feature design often depends on dataset and analyst hypotheses. However, deep learning methods utilize all pixel information in images, reducing the hypothesis dependence of feature design and extracting important features in a data‐driven manner. This approach offers a significant advantage by allowing exploration of crucial features in a bottom‐up manner rather than in a top‐down manner. In our study, we successfully identified: the eyes and nose are important for attractiveness evaluation, the eyebrow region for dominance evaluation and the region around the eyes for sex evaluation. However, to include correspondence between simplified features such as the width‐to‐height ratio of a face or the shape and size of the eyes, machine learning and deep learning features are expected to be integrated into the analysis.

Our approach considered both the face and the observer. Computational methods like geometric morphometrics and deep learning models effectively confirm factors on the observed face by aggregating impression ratings according to participant characteristics, while experimental methods consider observer information. We examined facial features extracted by geometric morphometrics and verified results with eye tracking and impression evaluation experiments. We combined deep learning with eye tracking to identify regions used by the deep learning model and gaze areas. Correlation analysis showed that while eye tracking and deep learning use different cues, both highlighted eyes, eyebrows and nose areas. Deep learning captures more facial features than visual attention alone, making it practical for comprehensive feature analysis.

Our study has several limitations. First, the number of original face images used was 290 male images and 307 female images; as such, our results may depend on the facial features of the face image set used. Currently, only a few face image datasets have been assigned facial impression scores owing to the controlled shooting conditions, and we expect that more extensive datasets will be released in the future. Second, because a simple CNN model was used in this study, there were limitations in the representation of this model. Although the human visual attention regions in this study were different from the predictor region cues in the deep learning model, advanced deep learning models that consider human perceptual characteristics will be developed in the future to extract more common features. Third, in Study 1, we conducted morphometric analysis based on the impression scores in the Chicago Face Database, which did not indicate the sex of the rater. Future work should include an analysis using impression scores for each rater sex. Finally, given our focus on morphological features and face images expanded by morphometrics, we could not conduct an in‐depth study of features related to skin and textures. In the analysis using the deep learning model in Study 3, detailed features were extracted from the skin region in addition to morphological features. To interpret these features in detail, further research is needed to verify the influence of morphological features and texture information by combining image statistical calculations.

## CONCLUSION

We used eye tracking and deep learning to search for influential facial features using face images generated using geometric morphometrics. The results revealed that while eye‐tracking and deep learning use different features as cues, deep learning effectively extracts a wide range of features that cannot be captured by visual attention alone. In the eye‐tracking experiments, visual attention was focused on salient facial features such as the eyes, nose and mouth. In the deep learning analysis, broader facial features such as eyebrows and superciliary archesc were also identified. The deep learning analysis also extracted differences in the details of these features by the rater's sex. The exploratory approach of this study using deep learning methods will likely contribute to the current understanding of detailed psychological findings. In addition, because of the rapid development of explainable AI techniques, such an approach will continue to be useful in face research in psychology, especially to examine detailed facial impression factors.

## AUTHOR CONTRIBUTIONS

**Takanori Sano:** Conceptualization; methodology; investigation; writing – original draft; validation; formal analysis; funding acquisition; project administration; visualization; writing – review and editing; data curation. **Jun Shi:** Investigation; writing – review and editing; formal analysis; visualization. **Hideaki Kawabata:** Conceptualization; funding acquisition; writing – review and editing; project administration; resources; supervision.

## CONFLICT OF INTEREST STATEMENT

The authors declare no competing interests.

## Supporting information


Appendix S1

## Data Availability

The created face image data, landmark point data, rated data, models and R code are available at https://osf.io/3rdmx/. The Grad‐CAM was implemented with reference to the example provided in the Keras documentation (https://keras.io/examples/vision/grad_cam/).
