# Supplementary material for: The differences in essential facial areas for impressions between humans and deep learning models: An eye‐tracking and explainable AI approach
Source: Br J Psychol. 2024 Oct 25;117(2):503–27. doi: 10.1111/bjop.12744 (PMC13051033; doi:10.1111/bjop.12744)
Supplement: Supplementary file 1 — Appendix S1 [file BJOP-117-503-s001.docx]

*British Journal of Psychology*

The Differences in Essential Facial Areas for Impressions Between Humans and Deep Learning Models: An Eye-Tracking and Explainable AI Approach

**STUDY 2: VERIFICATION BY EYE‐TRACKING MEASUREMENT**

**Analysis 1: Linear mixed-effects models for morphological changes and impression ratings**

**Results**

We conducted a mixed analysis of variance (ANOVA), two image sexes (male and female) × four image races (Asian, Black, Latin and White) × two data types (the data set of this study and the Chicago data base) design, to examine the effects on the evaluations of attractiveness, dominance and sexual dimorphism. Image sex and race were between-subjects factors, while data type was a within-subjects factor.

For attractiveness (Figure S1), there was no significant main effect of image sex (*F* (1, 72) = 2.371, *p* = 0.128, *η²* = 0.022), or of image race (*F* (3, 72) = 1.985, *p* = 0.124, *η²* = 0.054). However, the analysis revealed a significant main effect of data type (*F* (1, 72) = 6.14, *p* = 0.016, *η²* = 0.014.), and a significant interaction effect was observed between image sex and data type (*F* (1, 72) = 12.848, *p* < 0.001, *η²* = 0.030). We conducted a simple effects analysis to examine the significant image sex × data type interaction. The effect of data type was significant only at male of image sex (*F* (1, 36) = 21.965, *p* < .0001, *η²* = .0895). For the male images, the mean score from the present study (M = 3.502, *SD* = 0.688) was significantly higher than that from the Chicago data base (M = 3.073, *SD* = 0.698).

Furthermore, we examined whether attractiveness ratings of Asian participants change based on whether the face image is of the same or different race. To analyze this, we conducted an ANOVA with the factors of image sex and image race as between-subjects variables on the data collected. The analysis revealed a significant main effect of image race (*F* (3, 72) = 4.738, *p* = 0.005, *η²* = .156). No significant main effect was found for image sex (*F* (1, 72) = 0.082, *p* = 0.775, *η²* = .001). The interaction effect between image sex and race was not significant (*F* (3, 72) = 1.658, *p* = 0.184, *η²* = 0.055). For male images, Latin images (M = 3.853, *SD* = 0.514) were rated significantly higher than Asian images (M = 3.071, *SD* = 0.858). For female images, Latin images (M = 3.831, *SD* = 0.492) were rated significantly higher than Black images (M = 3.032, *SD* = 0.597).


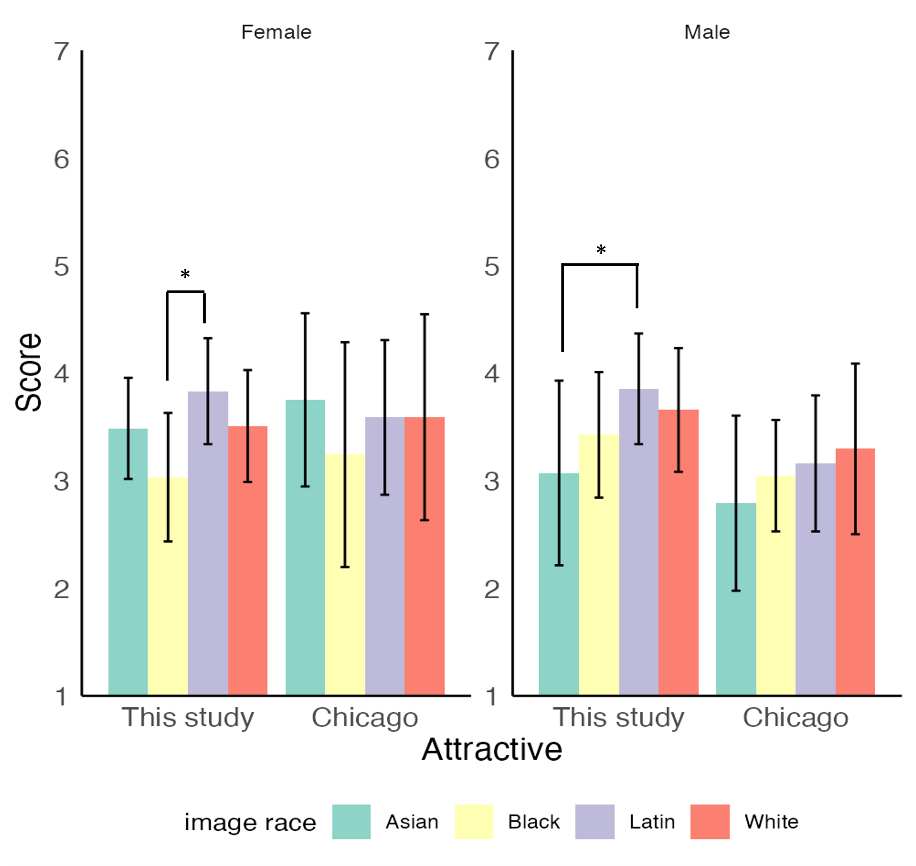


Figure S1: Attractiveness ratings across image sex and image race in this study and Chicago data base (*p* < 0.05: *, *p* < 0.01: **, *p* < 0.001: ***)

For dominance (Figure S2), image race had a significant main effect (*F (*3, 72) = 3.575, *p* = 0.018, *η²* = 0.042). Data type had a highly significant main effect (*F* (1, 72) = 119.539, *p* < 0.001, *η²* = 0.333). There was no significant main effect of image sex (*F* (1, 72) = 2.861, *p* = 0.095, *η²* = 0.011). The interaction between image sex and data type was significant (*F* (3, 72) = 3.50, *p* = .0197, *η²* = .0414). The interaction between image sex and data type was highly significant (*F* (1, 72) = 15.913, *p* < 0.001, *η²* = 0.044). The interaction between image race and data type was also significant (*F* (3, 72) = 5.054, *p* = 0.003, *η²* = 0.042). We conducted a simple effects analyses to examine the significant image sex × data type interaction. The effect of data type was significant at male images (*F* (1, 36) = 18.937, *p* < 0.001, *η²* = .162). For the male images, the mean score of dominance from the present study (M = 3.742, *SD* = 0.672) was significantly higher than that from the Chicago data base (M = 3.196, *SD* = 0.584). The effect of data type was significant at female images (*F* (1, 36) = 153.202, *p* < 0.001, *η²* = 0.541). For the female images, the mean score of dominance from the present study (M = 3.898, *SD* = 0.591) was significantly higher than that from the Chicago data base (M = 2.725, *SD* = 0.501).

Furthermore, we examined whether the dominance ratings of Asian participants change based on whether the face image is of the same or different race. We conducted an ANOVA with the factors of image sex and race as between-subjects variables on the data collected in this study. The analysis revealed a significant main effect of image race (*F* (3, 72) = 4.699, *p* = 0.005, *η²* = 0.150). No significant main effect was found for image sex (*F* (1, 72) = 1.439, *p* = 0.234, *η²* = 0.001). The interaction effect between image sex and image race was not significant (*F* (3, 72) = 2.261, *p* = 0.089, *η²* = 0.072).

For male images, there was no differences between image races (*F* (3, 72) = 1.112, *p* = 0.3500, *η²* = 0.035). For female images, Asian images (M = 4.217, *SD* = 0.582) were rated significantly higher than Latin images (M = 3.242, *SD* = 0.423) (*p* < .001). Black images (M = 4.089, *SD* = 0.408) were rated significantly higher than Latin images (M = 3.242, SD = 0.423) (*p* < .01). White images (M = 4.043, *SD* = 0.413) were rated significantly higher than Latin images (M = 3.241, *SD* = 0.423) (p < .01).


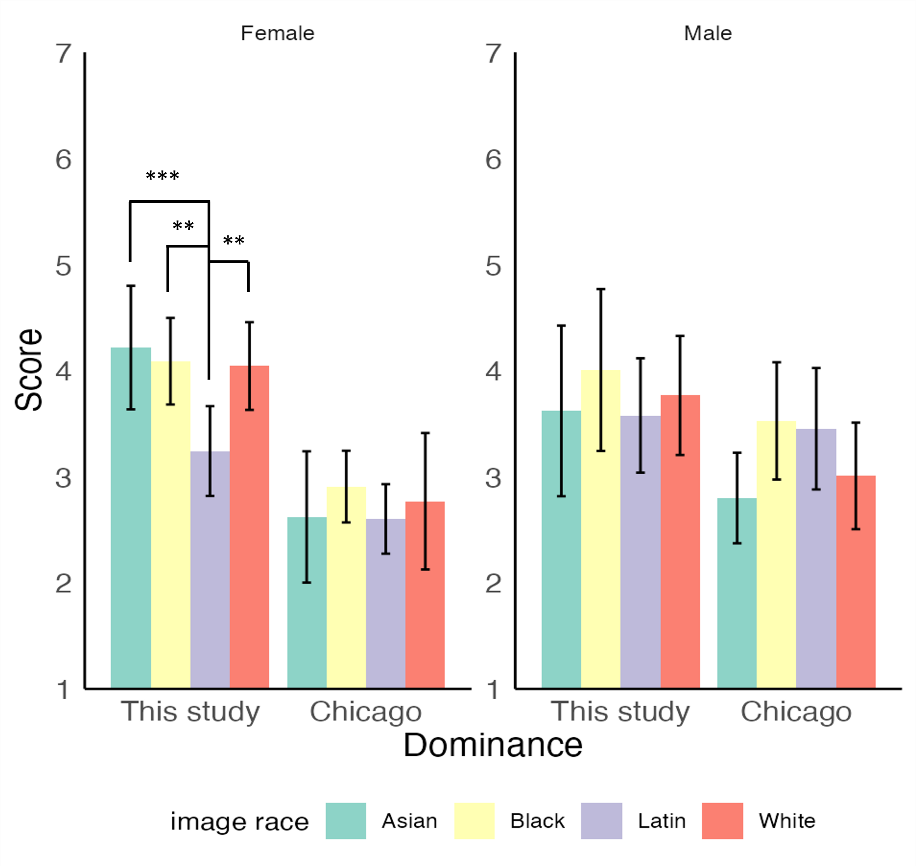


Figure S2: Dominance ratings across image sex and image race in this study and Chicago data base (*p* < 0.05: *, *p* < 0.01: **, *p* < 0.001: ***)

For sexual dimorphism (Figure S3), image sex had no significant main effect (*F* (3, 72) = 1.865, *p* = 0.176, *η²* = 0.013). There was no significant main effect of image race (*F* (3, 72) = 1.240, *p* = 0.302, *η²* = 0.026). Data type had a significant main effect (*F* (1, 72) = 7.393, *p* = .008, *η²* = 0.019). The interaction between image sex and image race was significant (*F* (3, 72) = 9.024, *p* < 0.001, *η²* = 0.189). The interaction between image sex and data type was not significant (*F* (1, 72) = 0.361, *p* = 0.550, *η²* = 0.001). The interaction between image race and data type was not significant (*F* (3, 72) = 1.294, *p* = 0.283, *η²* = 0.010).

Furthermore, we examined whether the sexual dimorphism evaluations of Asian participants change based on whether the face image is of the same race or a different race. We conducted an ANOVA with image and image race as between-subjects variables on the data collected in this study. The analysis revealed no significant main effect of image sex (*F* (1, 72) = 2.201, *p* = 0.142, *η²* = 0.019). No significant main effect was found for image race (*F* (3, 72) = 1.130, *p* = 0.342, *η²* = 0.029). The interaction effect between image sex and race was significant (*F* (3, 72) = 12.518, *p* < 0.001, *η²* = 0.326).

For male images, there was significant differences between image races (*F* (3, 72) = 5.128, *p* = 0.003, *η²* = 0.134). Black images (M = 5.080, *SD* = 0.546) were rated significantly higher than White images. For female images, there was significant differences between image races (*F* (3, 72) = 8.520, *p* < 0.001, *η²* = 0.222). Asian images (M = 4.511, *SD* = 0.533) were rated significantly higher than Black images (M = 3.646, *SD* = 0.577; *p* < .001). Latin images (M = 4.757, *SD* = 0.639) were rated significantly higher than Black images (M = 3.646, *SD* = 0.577; *p* < .001). White images (M = 4.631, *SD* = 0.639) were rated significantly higher than Black images (M = 4.757, *SD* = 0.639; *p* < .001).


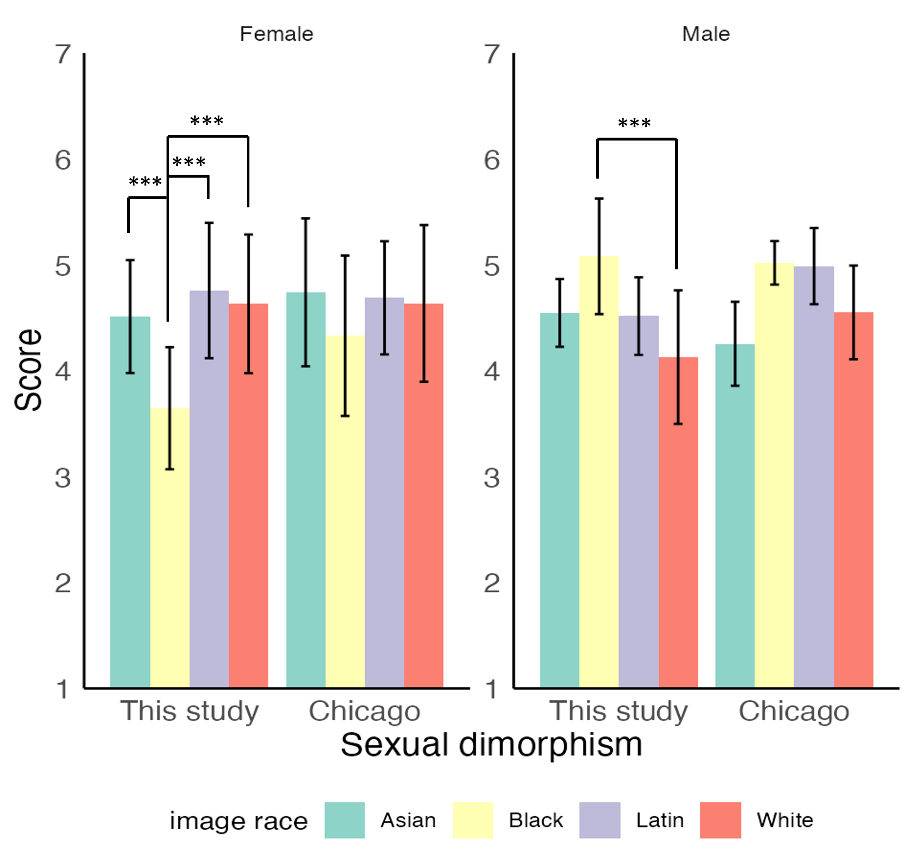


Figure S3: Sexual dimorphism ratings across image sex and image race in this study and Chicago data base (*p* < 0.05: *, *p* < 0.01: **, *p* < 0.001: ***)

**STUDY 3: ANALYSIS USING DEEP LEARNING METHODS**

**Analysis 2: Visualisation results with gradient-weighted class activation mapping**

**Results**

We confirmed the Grad-CAM heatmap for regions with values significantly greater than 0 by applying a false discovery rate (FDR) that accounts for type I errors due to multiple comparisons to a per-pixel t-test (p < .05). The figure on the left shows the average heatmap value for each image. In the figure on the right, white areas indicate significant pixel areas (Figure S4-6). The results showed that most of the areas were significant.


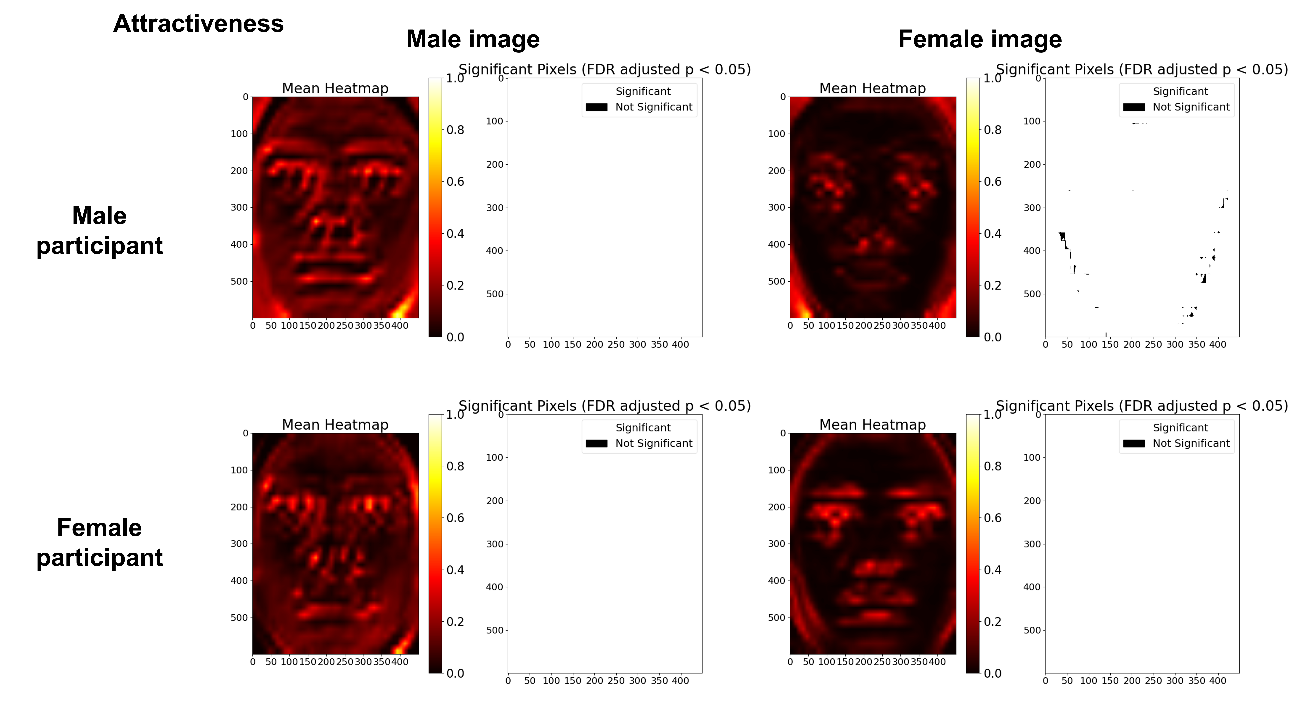


Figure S4 Significant areas of Grad-CAM heatmap in attractiveness


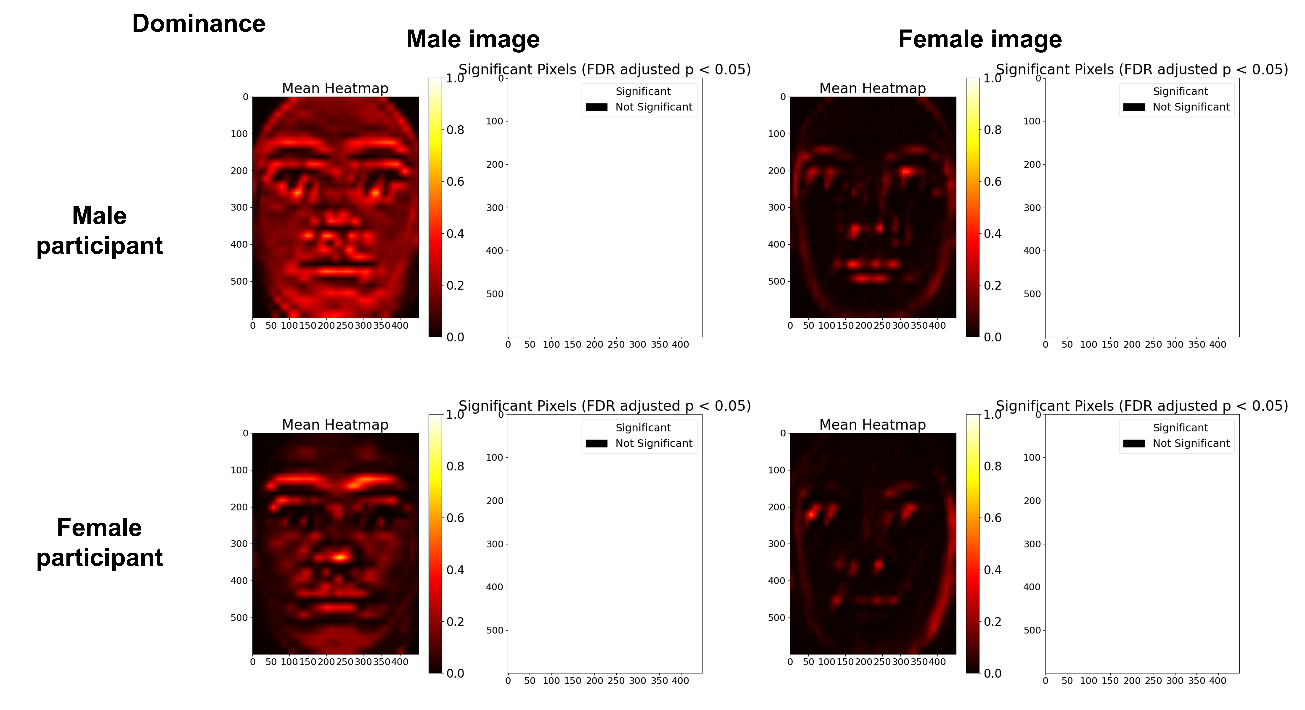


Figure S5 Significant areas of Grad-CAM heatmap in dominance


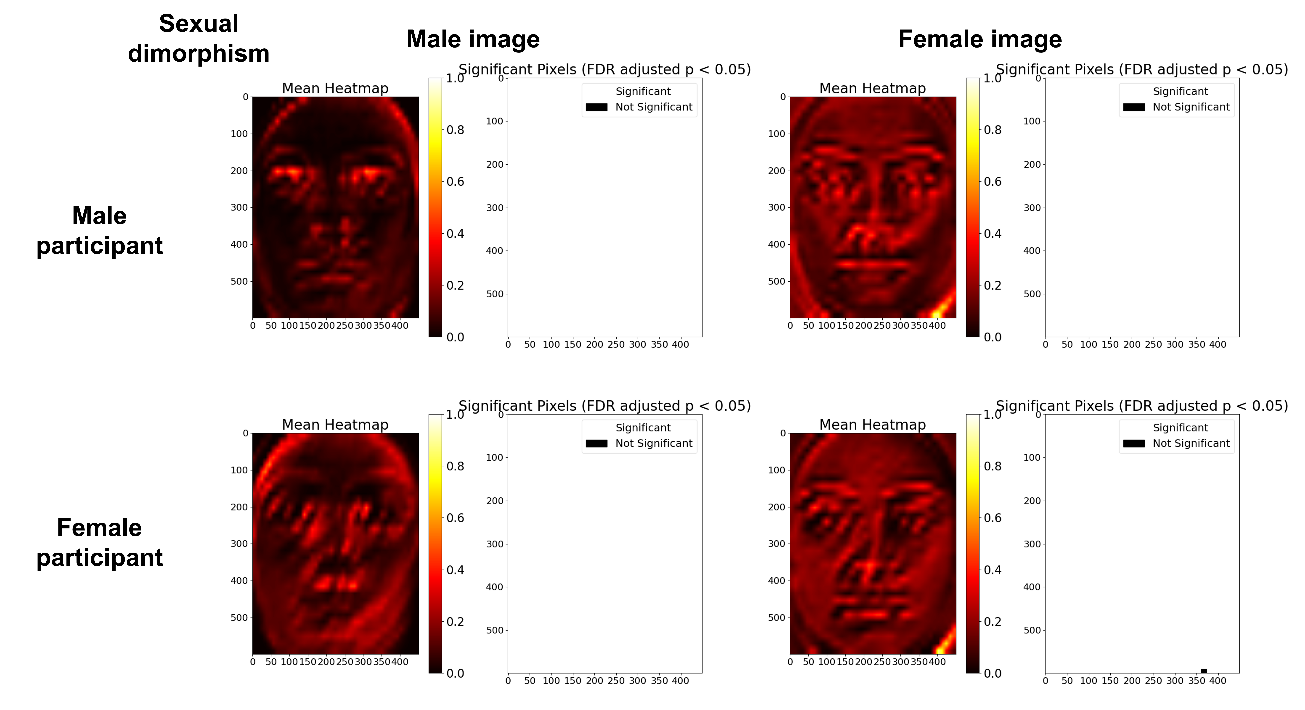


Figure S6 Significant areas of Grad-CAM heatmap in sexual dimorphism

**Analysis: Correlation analysis of heatmap values of the area of interest (AOI) for fixation time and Grad-CAM**

**Methods**

We performed a correlation analysis focusing on the area of interest (AOI) in the heatmap values extracted by Grad-CAM and the fixation time extracted in Study 2 to investigate whether the two techniques measure the same variables. We employed the limited-radius Voronoi-tessellation (Hessels et al., 2016) method for AOI and defined the eye, nose, and mouth regions using the proposed tool (Vehlen et al., 2022; Figure S7). We calculated the average fixation time and Grad-CAM values for these regions for each image and min-max normalised each to the range 0-1. We used the values for each AOI to create scatter plots of the fixation time and Grad-CAM values and performed a correlation analysis. We created scatter plots and performed correlation analysis by image and participant sex.


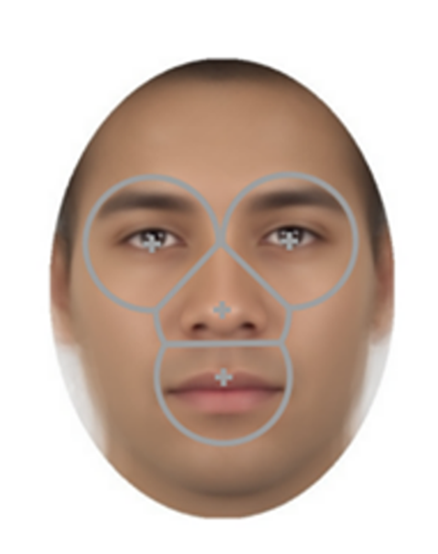


Figure S7: Example of the area of interest (AOI) for a face: The area was calculated using the limited-radius Voronoi-tessellation method. The Grad-CAM heatmap values and fixation time heatmap values for this region were extracted (see Figures S8-10).

**Results**

The results of the correlation analyses for attractiveness, dominance, and sexual dimorphism are shown in Figures S8, S9, and S10, respectively. Correlation coefficients and p-values are shown in the figures. Orange dots and lines indicate female images; blue dots and lines indicate male images. The thin line indicates the 95% confidence interval of the regression line. Results showed almost no correlation between Grad-CAM and fixation time for any impression and AOI. Thus, the results suggest that in the AOI region, there is no statistical relationship between the regions contributing to impression prediction extracted by deep learning and the human gazing region.


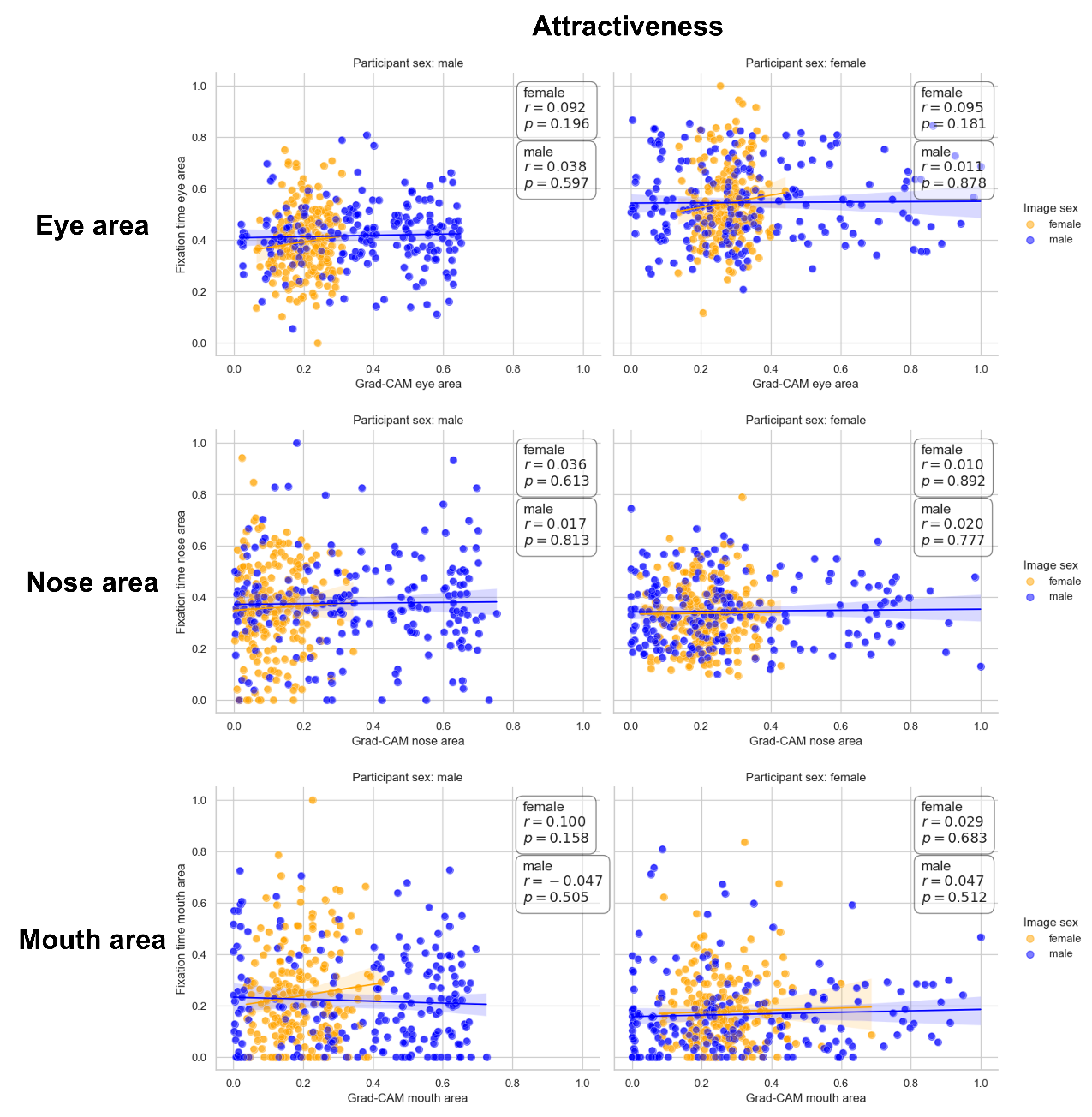


Figure S8: Scatter plots of heatmap values of Grad-CAM and fixation time for each AOI in attractiveness scores


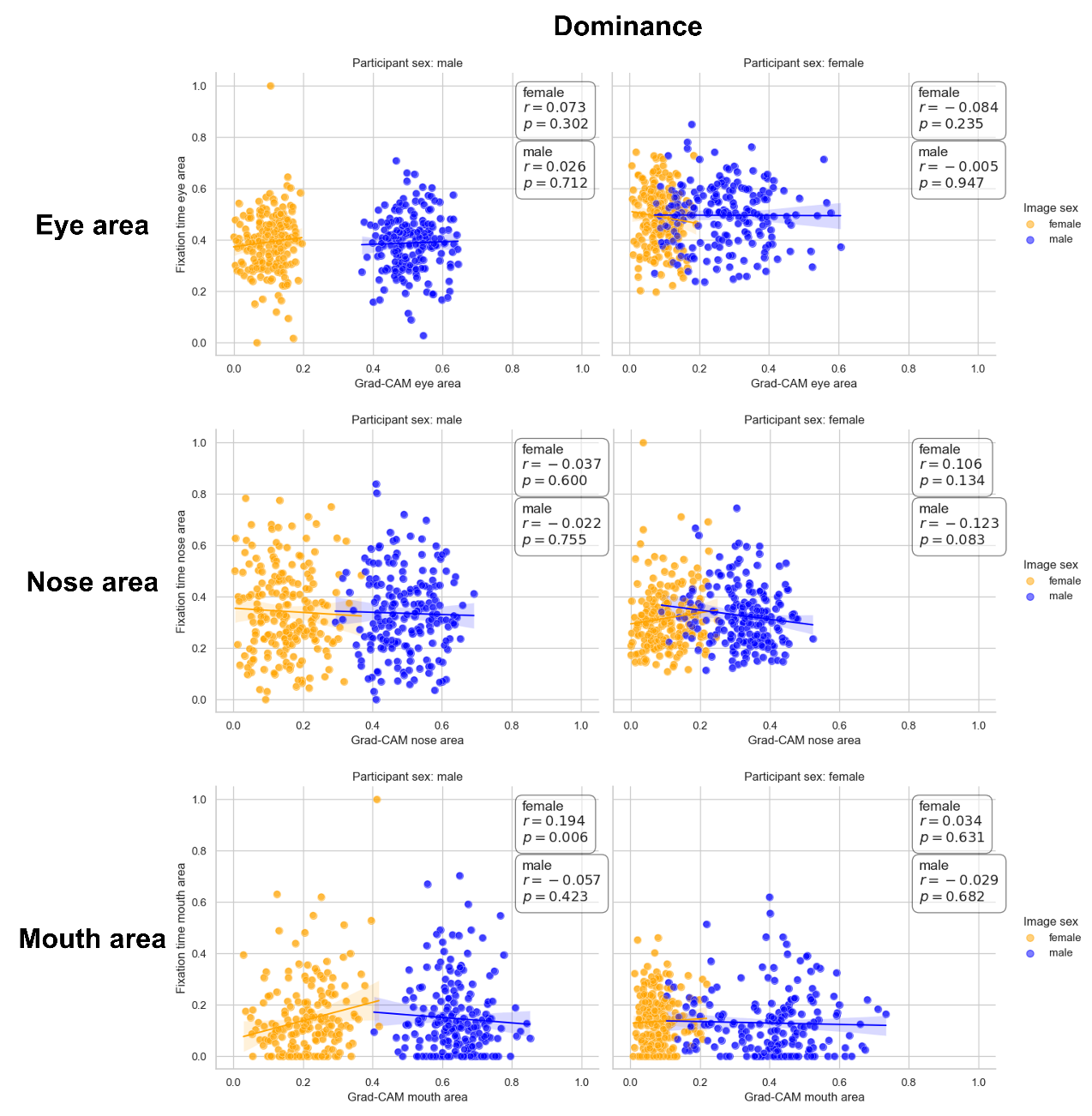


Figure S9: Scatter plots of heatmap values of Grad-CAM and fixation time for each AOI in attractiveness scores


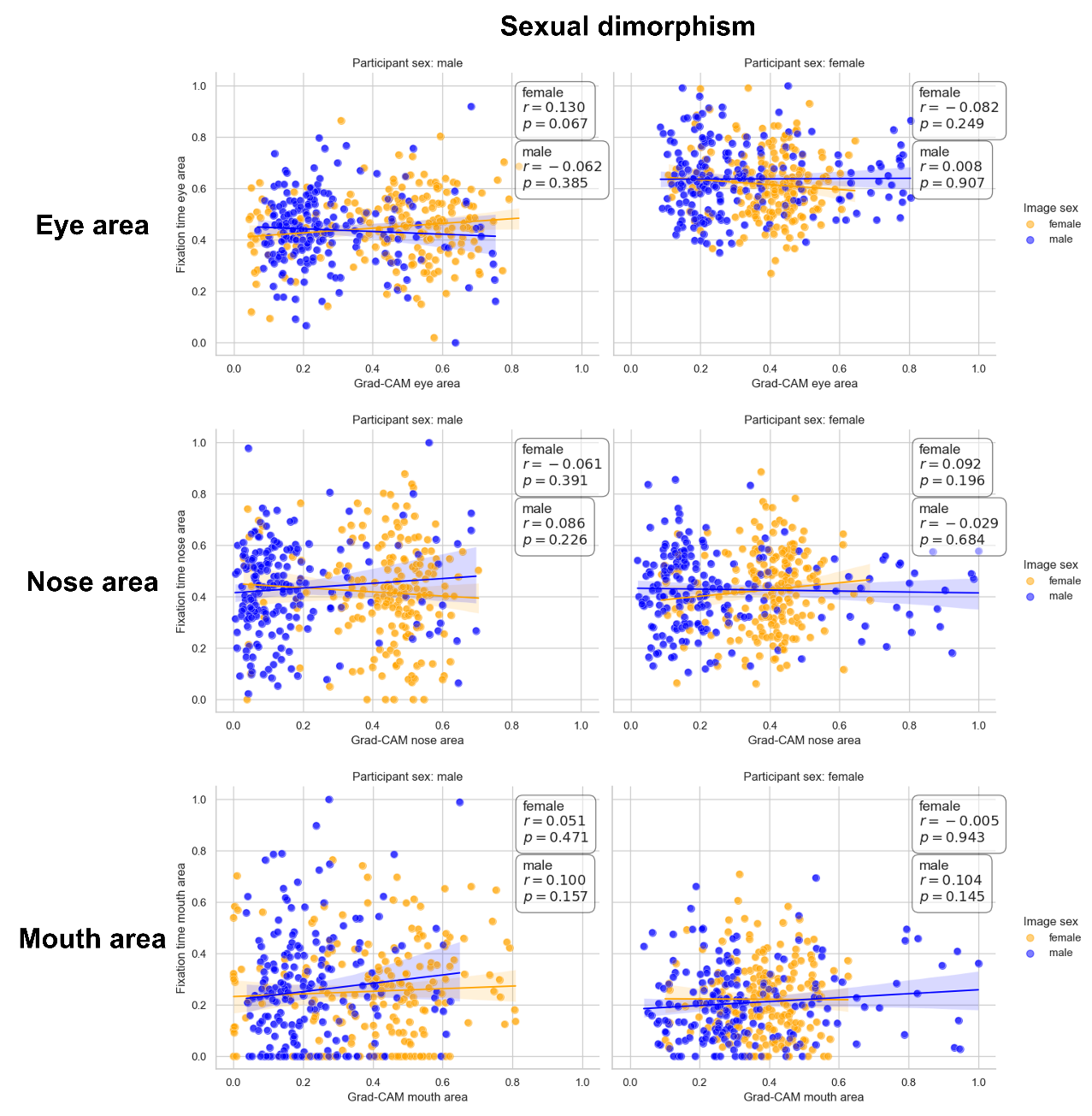


Figure S10: Scatter plots of heatmap values of Grad-CAM and fixation time for each AOI in attractiveness scores
